# *De novo* Assembly of Leaf Transcriptome in the Medicinal Plant *Andrographis paniculata*

**DOI:** 10.3389/fpls.2016.01203

**Published:** 2016-08-17

**Authors:** Neeraja Cherukupalli, Mayur Divate, Suresh R. Mittapelli, Venkateswara R. Khareedu, Dashavantha R. Vudem

**Affiliations:** Centre for Plant Molecular Biology, Osmania UniversityHyderabad, India

**Keywords:** *Andrographis paniculata*, *de novo* assembly, leaf transcriptome, terpenoid biosynthesis, cytochrome P450, simple sequence repeats

## Abstract

*Andrographis paniculata* is an important medicinal plant containing various bioactive terpenoids and flavonoids. Despite its importance in herbal medicine, no ready-to-use transcript sequence information of this plant is made available in the public data base, this study mainly deals with the sequencing of RNA from *A. paniculata* leaf using Illumina HiSeq™ 2000 platform followed by the *de novo* transcriptome assembly. A total of 189.22 million high quality paired reads were generated and 1,70,724 transcripts were predicted in the primary assembly. Secondary assembly generated a transcriptome size of ~88 Mb with 83,800 clustered transcripts. Based on the similarity searches against plant non-redundant protein database, gene ontology, and eukaryotic orthologous groups, 49,363 transcripts were annotated constituting upto 58.91% of the identified unigenes. Annotation of transcripts—using kyoto encyclopedia of genes and genomes database—revealed 5606 transcripts plausibly involved in 140 pathways including biosynthesis of terpenoids and other secondary metabolites. Transcription factor analysis showed 6767 unique transcripts belonging to 97 different transcription factor families. A total number of 124 CYP450 transcripts belonging to seven divergent clans have been identified. Transcriptome revealed 146 different transcripts coding for enzymes involved in the biosynthesis of terpenoids of which 35 contained terpene synthase motifs. This study also revealed 32,341 simple sequence repeats (SSRs) in 23,168 transcripts. Assembled sequences of transcriptome of *A. paniculata* generated in this study are made available, for the first time, in the TSA database, which provides useful information for functional and comparative genomic analysis besides identification of key enzymes involved in the various pathways of secondary metabolism.

## Introduction

Ever increasing interest in the development of novel drugs/drug lead molecules from phytochemicals with potential pharmacological and therapeutic activities—having minimum or no side effects—resulted in focused research on the plants used in traditional medicine. Vast majority of the species of genus *Andrographis* (Family Acanthaceae) were found to have medicinal value and are being used in the folk-medicine. Of all the species, *Andrographis paniculata* is being used extensively in Chinese and Indian medicine. It is widely distributed in the peninsular India and is commonly known as “Nelavemu or Kalmegh” (Neeraja et al., 2015). It grows as a herb in moist shady places and is being used in the treatment of various diseases. Phytochemical evaluation of *A. paniculata* revealed more than 20 diterpenoids and over 10 flavonoides (Tang and Eisenbrandt, 1992; Li et al., 2007). Andrographolide, a bicyclic diterpene lactone the principal secondary metabolite, isolated from the leaves and stem of *A. paniculata*, exhibited a broad range of pharmacological activities (Nugroho et al., 2012). Different semi-synthetic analog of andrographolide (Nanduri et al., 2004) have been produced on large scale and used extensively in various clinical applications as it is one of the safe natural drugs with multiple health promoting effects.

It was envisaged that the biosynthesis of andrographolide occurs through cytosolic mevalonate (MVA) and plastid-mediated methyl-D-erythritol 4-phosphate/Deoxy xylulose (MEP/DXP) pathways (Srivastava and Akhila, 2010). However, the key enzymes regulating the andrographolide synthesis remain unknown. Different plant genomes were known to contain gene families that code for enzymes with similar substrate-specificity, resulting in the formation of diverse products. Investigations indicated the existence of more than 25,000 terpenes across plant kingdom (McCaskill and Croteau, 1997), and the presence of various terpene synthases (TPSs) is responsible for the synthesis of molecules with diverse structures. High throughput Next Generation Sequencing (NGS) of transcriptomes of non-model organisms has revolutionized the functional genomics research. Recent studies with *Curcuma longa* (Annadurai et al., 2013), *Withania somnifera* (Dasgupta et al., 2014), *Camelina sativa* (Mudalkar et al., 2014), and *Bemisia tabaci* (Xie et al., 2012) have demonstrated the effectiveness of *de novo* assembly of eukaryotic transcriptomes.

Recently, transcriptome of *A. paniculata* was reported by Garg et al. (2015) and made available raw data as SRA files for root (SRX655521) and leaf (SRX652837) transcriptome. However, the assembled transcript sequences required by the researchers were not provided in any of the public data base. Hence, in this investigation, NGS technology has been used for RNA sequencing and *de novo* transcriptome assembly of *A. paniculata* leaf, using Illumina HiSeq™ 2000 platform, to identify known and novel transcripts of various metabolic pathways including terpenoid biosynthesis. Further, the study also focused on the identification of different cytochrome P450s expressed in the leaf, and the deduced proteins were classified into families. The assembled transcripts of this plant have been made available, for the first time, in the public data base as a TSA record. The assembled and annotated transcripts of *A. paniculata* can be used as the public information dataset.

## Materials and methods

### Plant material and RNA isolation

Seeds of *A. paniculata*, genotype Megha were collected from Central Institute of Medicinal and Aromatic Plants (CIMAP), Hyderabad. Plants were grown in the net house and fully expanded leaves from 3 months old potted plant were collected for RNA isolation. Leaves were dipped in liquid nitrogen and ground into fine powder using mortar and pestle. Total RNA was isolated according to manufacturer's protocol using Spectrum™ Plant Total RNA isolation kit from Sigma (Cat# STRN10). Agilent 2100 Bioanalyzer was used for determining RNA integrity number (RIN) and Nanodrop was used for RNA quantification.

### Generation of cDNA library and sequencing of transcriptome

RNA sample with RIN value more than seven was used for sequencing. Enriched poly-adenylated mRNAs using Oligo(dT) beads and were cut into short fragments. These fragments served as templates and random primers with a length of six nucleotides were used to synthesize cDNA by using reverse transcriptase. Double-stranded cDNA was further fragmented using DNase I to generate small fragments. These short fragments of double-strand cDNA were purified with QiaQuick PCR extraction kit. These fragments were connected with sequencing adapters and purified by agarose gel electrophoresis. Fragments ligated with adapters were used as templates for PCR amplification. DNA concentration of the sequencing library was determined on Agilent Bioanalyzer. The cDNA library was sequenced on the Illumina HiSeq™ 2000 platform in a Paired End 100 base run, using TruSeq PE Cluster Kit v3-cBot-HS (Catalog No.: PE-401-3001) for cluster generation on C-bot and TruSeq SBS Kit v3-HS (Catalog No.: FC-401-3001) for sequencing.

### *De novo* assembly of transcriptome

Output of raw reads of sequencing were subjected to stringent filtering conditions for the removal of reads with adaptors, reads with unknown nucleotides greater than 5% and reads with low quality. High quality (HQ) reads having more than 70% HQ bases (i.e., each base having ≥20 phred score) were considered to build up transcriptome. Primary assembly was carried out by merging the HQ reads using “Trinity” assembler (Grabherr et al., 2011) with a minimum contig length of 200 bases and k-mer size of 25 bp. A minimum count of 2 k-mers were assembled by Inchworm algorithm and a minimum number of 5 reads were used to glue two Inchworm contigs together. In order to cluster contigs originating from the same gene or protein, a secondary assembly was carried out using CD-HIT EST (v4.6.1) tool (Li and Godzik, 2006). Homologous contigs with 80% identity were clustered to generate full length transcripts. The secondary assembly was evaluated by mapping HQ paired end reads to clustered trancscripts using Bowtie2 (Liu and Schmidt, 2012). The sequence data generated in this study have been deposited at NCBI in the Short Read Archive database under the accession number SRX544977 (link: http://www.ncbi.nlm.nih.gov/sra/?term=SRX544977) (Bioproject ID: PRJNA247458, Biosample ID: SAMN02777339). All the assembled transcript sequences were also deposited in DDBJ/EMBL/GenBank under the accession GBJB00000000 as Transcriptome Shotgun Assembly (TSA) project and the individual sequences are available in the TSA master record GBJB00000000.

### Functional annotation and biological classification of transcripts

Functional annotation and classification of assembled transcripts was done using Blast2GO tool (*E* ≤ 10^−2^, Annotation cutoff–55, GO weight–5,) against plant Non-Redundant protein Data Base (NRDB) Kyoto Encyclopedia of Genes and Genomes (KEGG) pathway (http://www.genome.jp/kegg/), Eukaryotic Orthologous Groups (KOG) (http://genome.jgi-psf.org/help/kogbrowser.jsf), and Gene Ontology (GO) using NCBI nr database (http://www.ncbi.nlm.nih.gov). Corresponding GO IDs were obtained using NCBI accession number and AmiGO2 is used to obtain GO description for GO IDs (http://amigo.geneontology.org/amigo). The best aligned transcripts were used to decide the sequence direction and retrieve proteins with the highest sequence similarity. Pathway assignment for the annotated transcripts was carried out using KEGG mapping (http://www.genome.ad.jp/kegg/). KEGG orthologs were identified using the KEGG Automated Annotation Server (KAAS) with default parameters. Unannotated transcripts were subjected to Conserved Domain Database (CDD) for the identification of possible functional domains (http://www.ncbi.nlm.nih.gov/Structure/cdd/cdd.shtml).

### Identification of transcription factors

Transcription factors (TFs) were identified using genome-scale protein and nucleic acid sequences by analyzing InterProScan domain patterns in protein sequences with high coverage and sensitivity using PlantTFcat analysis tool (http://plantgrn.noble.org/PlantTFcat/) (Dai et al., 2013).

### Identification and classification of Cytochrome P450 (CYP450) proteins

*A. paniculata* transcriptome deduced proteome was searched and putative CYPs were retrieved. Naming of *A. paniculata* CYPs was carried out based on similarity to the available plant CYPs subjecting to BLASTP analysis (http://drnelson.uthsc.edu/CytochromeP450.html) (Nelson et al., 2008). Further, the CYPs were confirmed by the presence of motif using ExPASy–PROSITE tool (http://prosite.expasy.org/). Multiple sequence alignment of CYP proteins was performed using the UPGMB clustering (Gap opening −2.9 and gap extension penalty 0), in the MUSCLE module (Edgar, 2004) from the Mega 5.2.2 software. Phylogenetic tree was generated using Maximum Likelihood (ML) method in MEGA 5.2.2 (Hall, 2013). The level of significance for the ML analysis of phylogenetic tree was carried out with bootstrap testing using 1000 replications (Kumar et al., 2015).

### Identification of simple sequence repeats (SSRs)

Simple sequence repeats were identified using MIcroSAtellite identification tool v1.0 (MISA) (http://pgrc.ipk-gatersleben.de/misa/). Unit size cut off of 6 was used to consider a di-nucleotide repeat and 5 for SSRs of 3-, 4-, 5,- and 6-nucleotide repeats. Maximum of 100 interrupting bases were allowed between 2 SSRs in a compound microsatellite.

### Quantitative real time polymerase chain reaction (qRT-PCR) of selected transcripts

Total RNA was isolated from the leaves of *A.paniculata* using trizol reagent (Cat# 15596-018) according to manufacturer's protocol. The first strand cDNA was synthesized from 5 μg of total RNA by using Oligo(dT) and SuperScript III Reverse Transcriptase (Invitrogen, Cat. No.18080-051). Transcripts encoding Cytochrome P450s viz., CYP94B56, CYP96A96, CYP96A97 and CYP94B, two enzymes involved in terpenoid biosynthesis 4-hydroxy-3-methylbut-2-enyl diphosphate reductase (HMDR) and 2-C-methyl-D-erythritol 4-phosphate cytidylyltransferase (MED) and house-keeping transcript actin were picked from the annotated sequences of the transcriptome. Transcript specific primers (Supplementary File S1) were designed and PCR based expression profiling was carried out for each transcript in triplicates. A total volume of 20 μL reactions were setup for qRT-PCR using KAPA SYBR FAST Universal qPCR Kit (Cat# 51230-100) (9.8 μL of 2X reaction buffer, 0.2 μL of Kapa SYBR Green, 2.0 μL of cDNA, 2.5 pmol/μL of each forward and reverse primers and 6 μL of water). The relative expressions of six transcripts were calculated by taking the mean cycle threshold (Ct) values against mean Ct value of actin. Relative expression ratio was calculated using the formula 1/2^[Ct(actin) − Ct(gene)]^, where 2 represents perfect PCR efficiency.

## Results

### Paired end sequencing of cDNA library and *de novo* assembly of transcriptome

**Figure 1:**
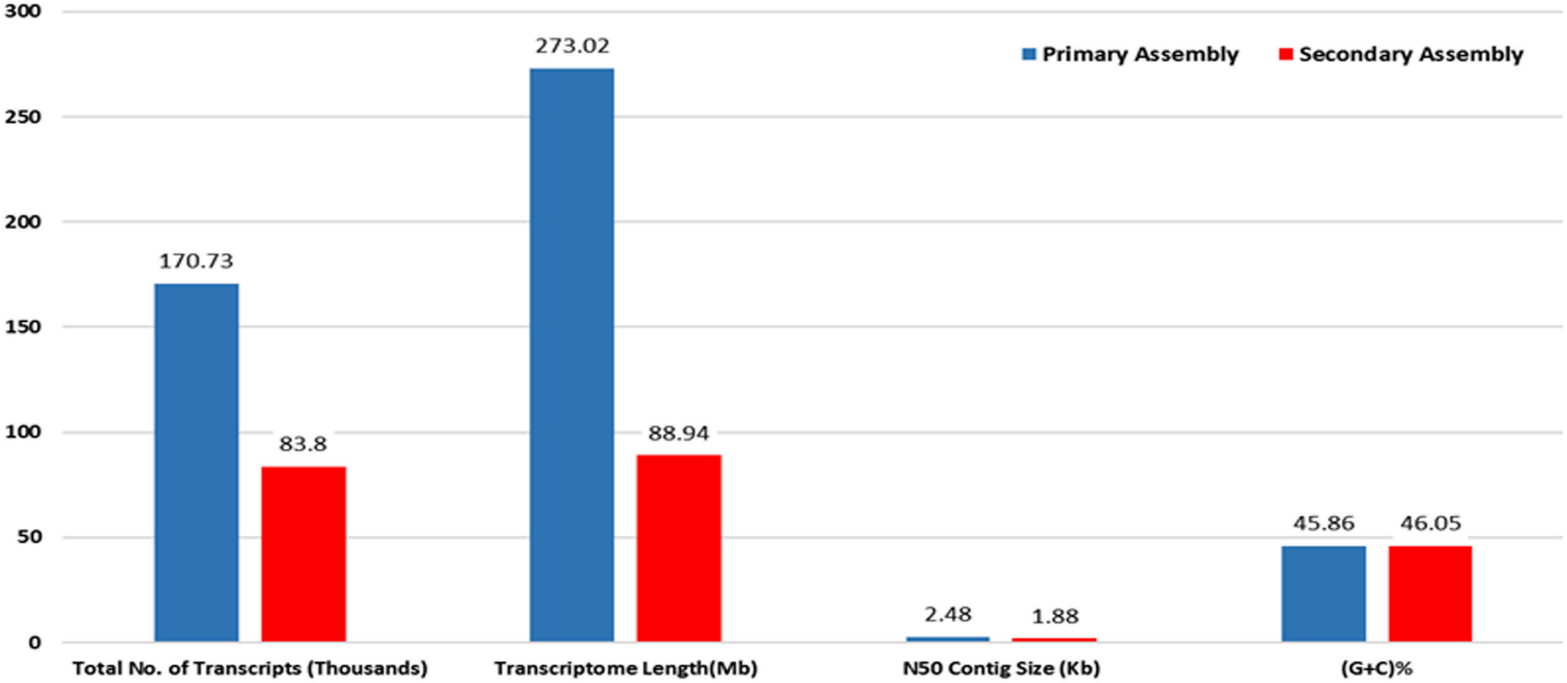
**Statistics of primary and secondary assembly of ***A. paniculata*** leaf transcriptome**.

**Figure 2:**
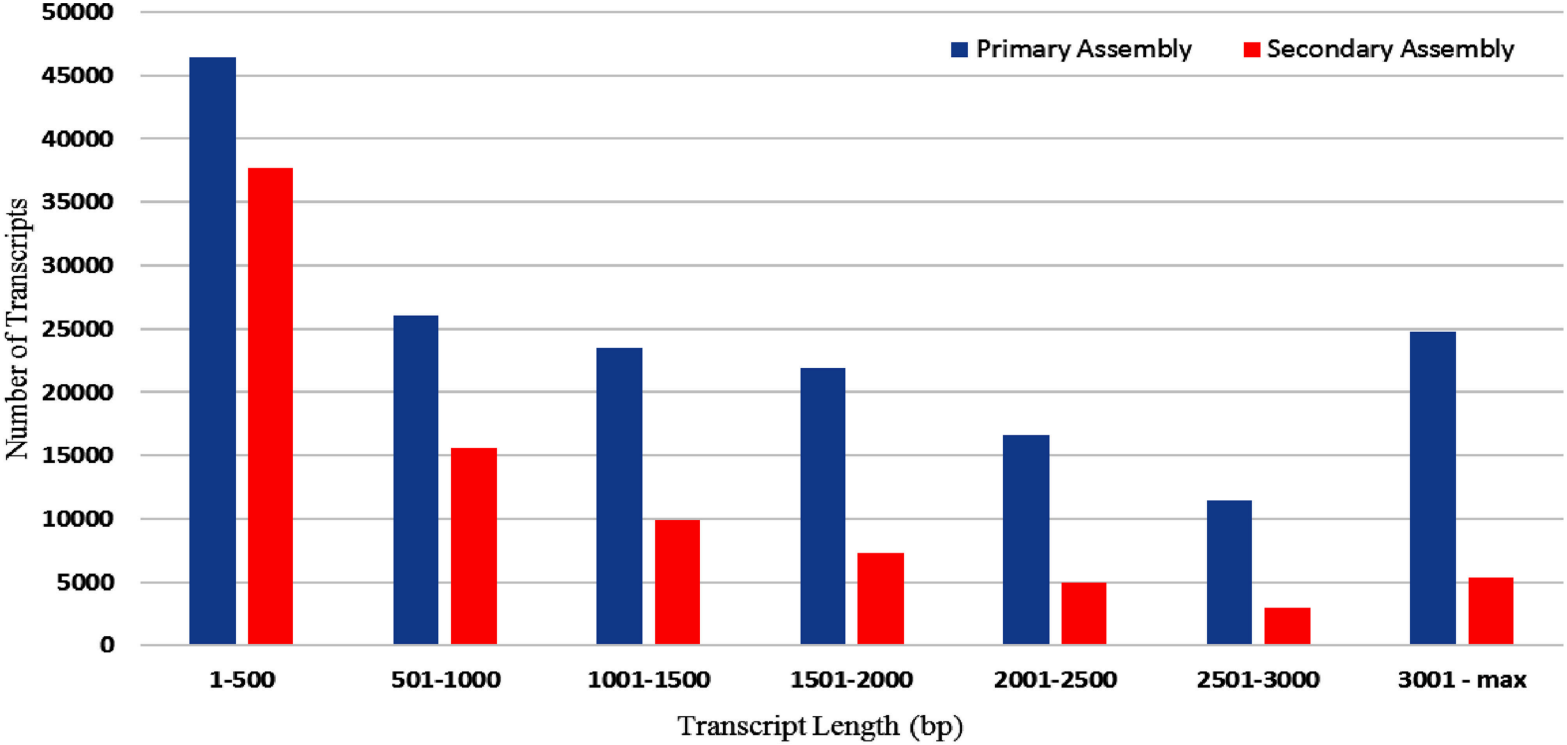
**Size distribution of ***A. paniculata*** leaf transcripts (bp) obtained both in primary and secondary assembly**.

RNA sample used in the present study was of good quality with RIN value more than 7. Paired end sequencing of the cDNA library, using Illumina HiSeq™ 2000, generated millions of reads with a length of 100 bp. After the removal of adapter sequences and low quality reads with phred score <20, a total of 189.22 million high quality clean reads were obtained representing 95.8% of the transcriptome. Primary assembly of high quality reads using Trinity assembler produced a total of 1,70,724 (~273 Mb) transcripts with minimum and maximum length being 201 and 13,383 bp, respectively. An average of 1599 bp of transcript length with an N50 of 2479 bp was identified. Primary assembly of transcriptome revealed 45.86% of GC content. Secondary assembly using CD-HIT-EST (v4.6.1) tool with 80% identity threshold, generated a total number of 83,800 clustered transcripts, constituting the transcriptome size of ~89 Mb with an N50 value of 1880 bp (Figure 1) and an average transcript length being 1061 bp. Evaluation of secondary assembly by mapping the high quality paired end reads to clustered transcripts showed up to 96% coverage. Of the 83,800 clustered transcripts obtained in the secondary assembly, majority (37,655) of them were found between 201 and 500 bp followed by 15,623 transcripts in the range of 501–1000 bp. Further, it was observed that 9890 transcripts were in the range of 1001–1500 bp, 7370 transcripts in the range of 1501–2000 bp, 5357 transcripts in the range of 3001–13,383 bp, 4912 transcripts in the range of 2001–2500 bp and 3001 transcripts in the range of 2501–3000 bp (Figure 2).

### Functional annotation and classification of the clustered transcripts

**Figure 3:**
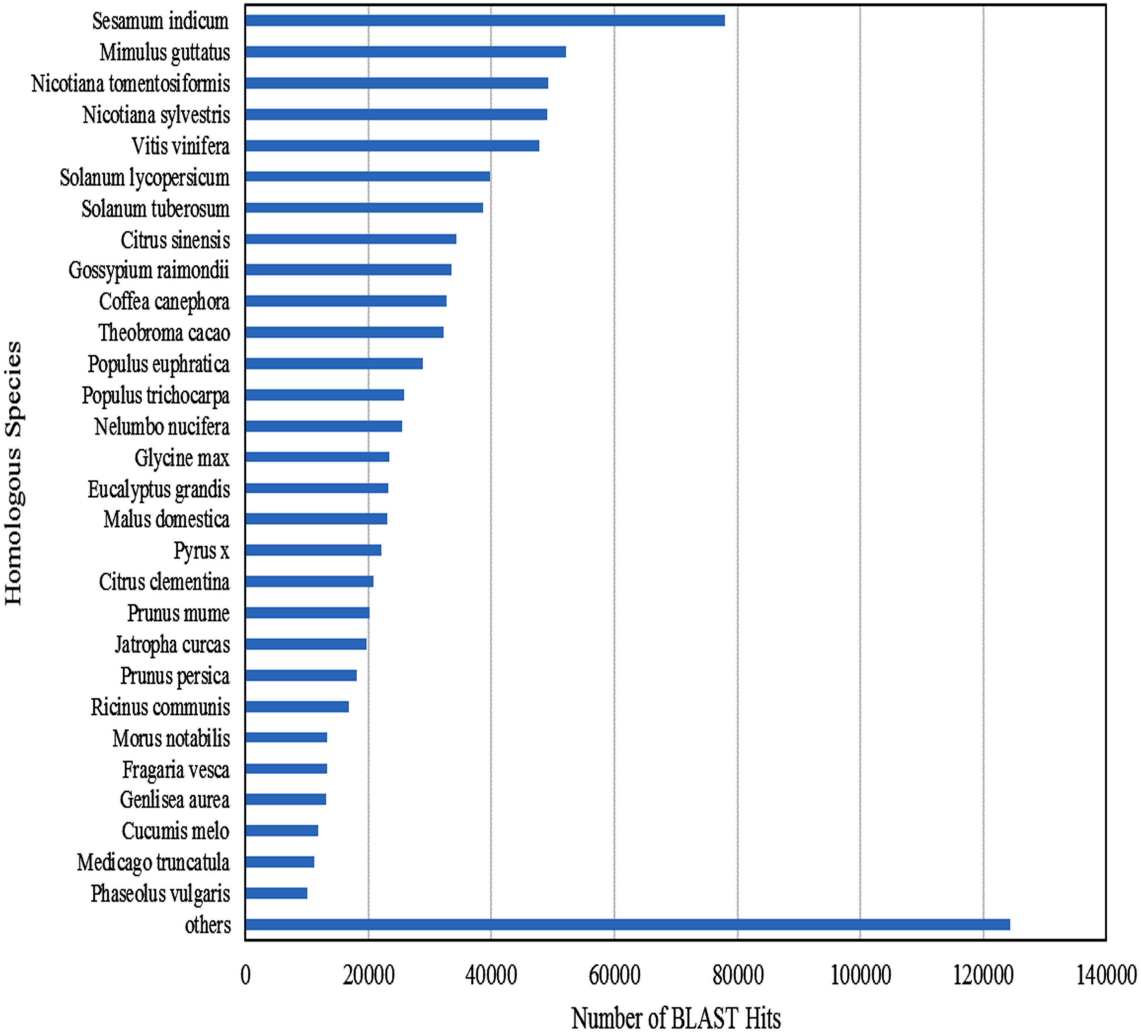
**Homologous species contributing for the functional annotation of clustered transcripts of ***A. paniculata*** leaf**.

Functional annotation of 83,800 clustered transcripts, using Blast2GO against various databases like Plant NRDB, GO, and KOG, resulted in 49,363 annotated transcripts constituting up to 58.91% with 30,677 unique transcripts. Functional annotations of the assembled transcripts with plant NRDB revealed that majority of them are homologous to the species *Sesamum indicum, Mimulus guttatus, Nicotiana tomentosiformis, Nicotiana sylvestris* etc (Figure 3).The remaining 34,437 unannotated transcripts when subjected to functional domain search against Conserved Domain Database (CDD), using RPS blast program with an *E*-value cutoff of 0.01, resulted in the annotation of 1961 additional transcripts constituting upto 5.69% and mapped with 16 unique domains (Supplementary File S2). Functional search for CDD domains identified majority of the transcripts (975) mapped to PRK12270 followed by COG3889, pfam11593, PTZ00436, PRK02888, PRK07003, PRK12678, PRK11907, PRK07735, and PRK09510 domains falling in the top 10 category.

### Annotation with gene ontology (GO)

**Figure 4:**
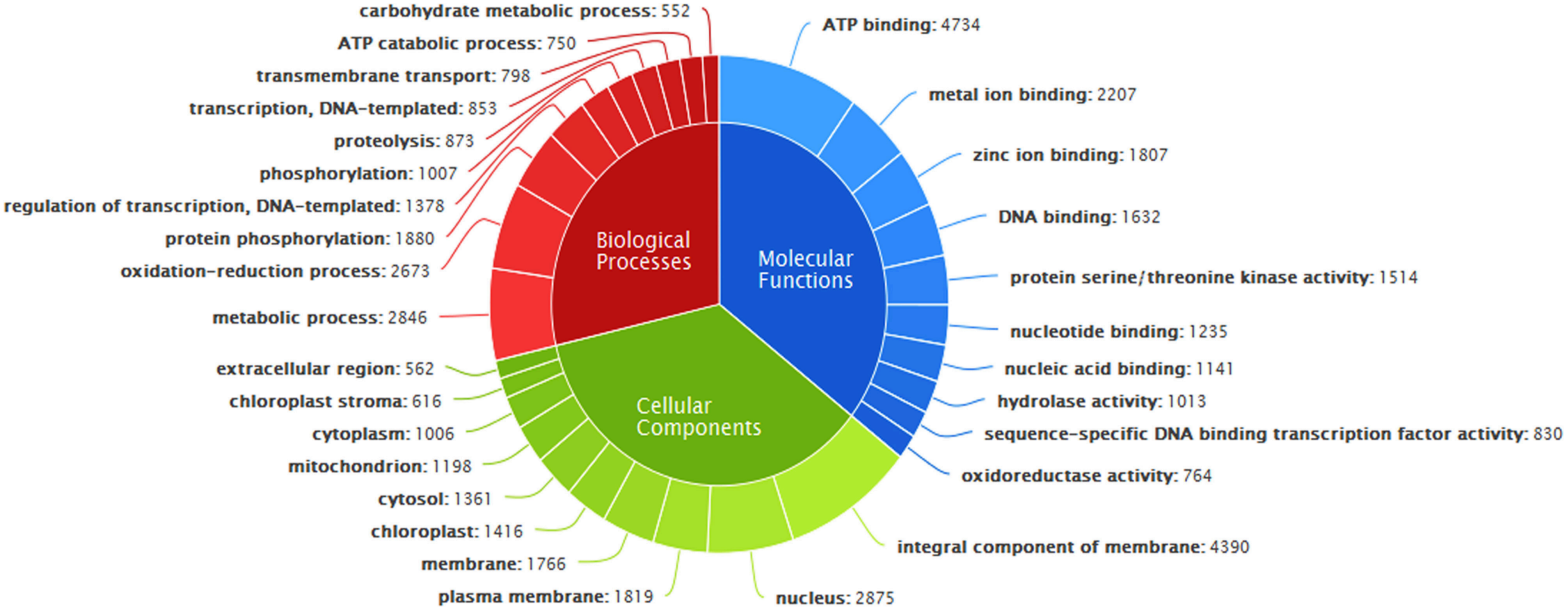
**Classification of ***A. paniculata*** leaf transcripts annotated with Gene Ontology**.

GO annotation of transcripts, based on sequence homology, classified them into three major functional categories corresponding to biological processes, cellular components, and molecular functions (Figure 4). Out of 49,363 annotated transcripts, 33,846 transcripts were predicted with gene ontology annotation. Overall, 4804 transcripts were assigned with unique GO terms. A total of 2415 transcripts were found in the category of biological processes with majority belonging to metabolic processes, oxidation-reduction processes, protein phosphorylation and regulation of transcription etc. About 32.98% of transcripts were mapped to different molecular functions with majority of them belonging to ATP binding followed by metal ion binding, zinc ion binding, DNA binding, protein serine/threonine kinase activity etc and the remaining transcripts were mapped to cellular components belonging to membranes of cell, nucleus, mitochondrion, plastids etc (Supplementary File S3).

### KOG analysis

KOG database was used to identify the functional characteristics of assembled transcripts by aligning them with an e-value cutoff, ≤0.01 and percentage identity of ≥30%. Out of 83,800 input transcripts, 35,401 transcripts were annotated of which 14,720 were found to be unique KOG mapped transcripts. KOG mapped transcripts were broadly classified into 25 different categories. Most of the transcripts (910) belonged to signal transduction mechanisms followed by 799 transcripts involved in post translational modification, protein turn-over chaperones for cellular processes and signaling and 548 transcripts were related to carbohydrate transport and metabolism (Supplementary Figure 1). Majority of the KOG mapped transcripts (17,168) were found homologous to the species *A. thaliana*. A total of 4796 unique transcripts were assigned with KOG IDs along with the respective enzyme codes (Supplementary File S4).

### Metabolic pathway analysis by KEGG

**Figure 5:**
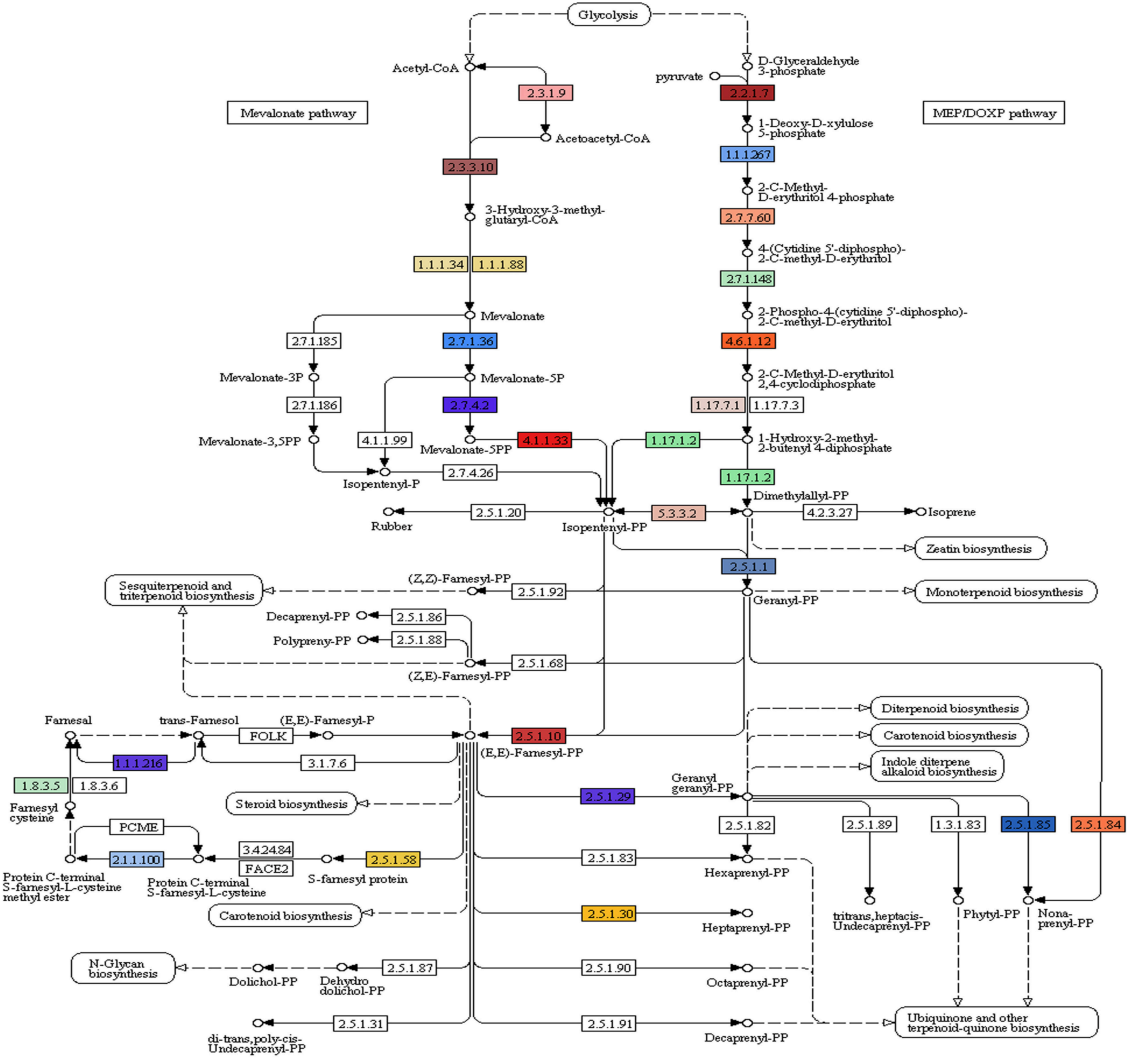
*****A. paniculata*** transcriptome encoded enzymes (highlighted) involved in terpenoid backbone biosynthesis**.

**Figure 6:**
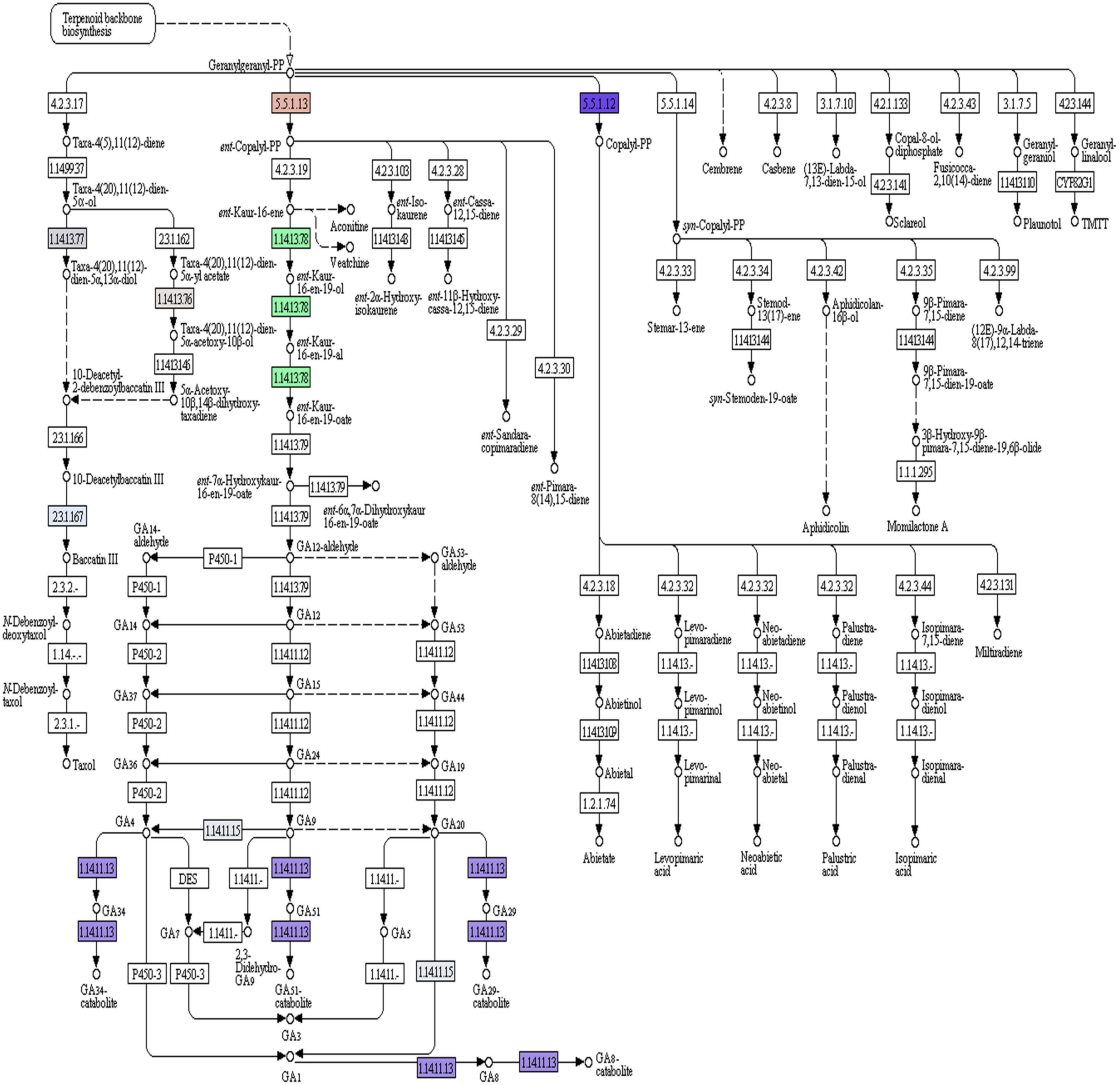
*****A. paniculata*** transcriptome encoded enzymes (highlighted) pertaining to di-terpenoid biosynthetic pathway**.

Of the 49,393 annotated transcripts, KEGG analysis provided functional classification and pathway assignment for 5606 transcripts. Interestingly, a high number (666) of identified transcripts were mapped to starch and sucrose metabolism, followed by 639 transcripts to purine metabolism, 297 transcripts to the category of pyramidine metabolism and 284 transcripts to the category of pentose and glucuronate interconversions (Supplementary File S5). Focus on mapping of the transcripts involved in the isoprenoid biosynthetic pathways identified 216 transcripts with a large number (96) of them being involved in terpenoid backbone biosynthesis followed by ubiquinone and other terpenoid-quinone biosynthesis (59), Diterpenoid biosynthesis (24), Monoterpenoid biosynthesis (21), while 13 transcripts were found to be involved in sesquiterpenoid and triterpenoid biosynthesis (Figures 5, 6).

### Transcripts encoding for the enzymes involved in the biosynthesis of terpenoids

*A. paniculata* leaf transcriptome revealed 146 different transcripts coding for enzymes involved in the biosynthesis of terpenoids (Supplementary File S6). Enzymes of terpenoid biosynthetic pathway displayed a wide variability of amino acids ranging from 66 to 1700 with the pI of 4.64 to 12.16. Many of these enzymes showed iso-forms and localized to the cytoplasm/chloroplast. Among the 146 enzymes, only 35 of them possessed motifs specific to terpene synthases. Fourteen terpene synthases contained DDXXD motif, seven with EDXXD motif, three with DXDD motif, three with DDYDE motif, two with DDXXD and DXDD motifs and, one contained DDXXD and EDXXD motifs. The remaining five enzymes revealed the presence of single modified motif, DIQDD/DDLVID/DEFDD/EDDDE/DARRDD.

### Transcription factor (TF) analysis

**Figure 7:**
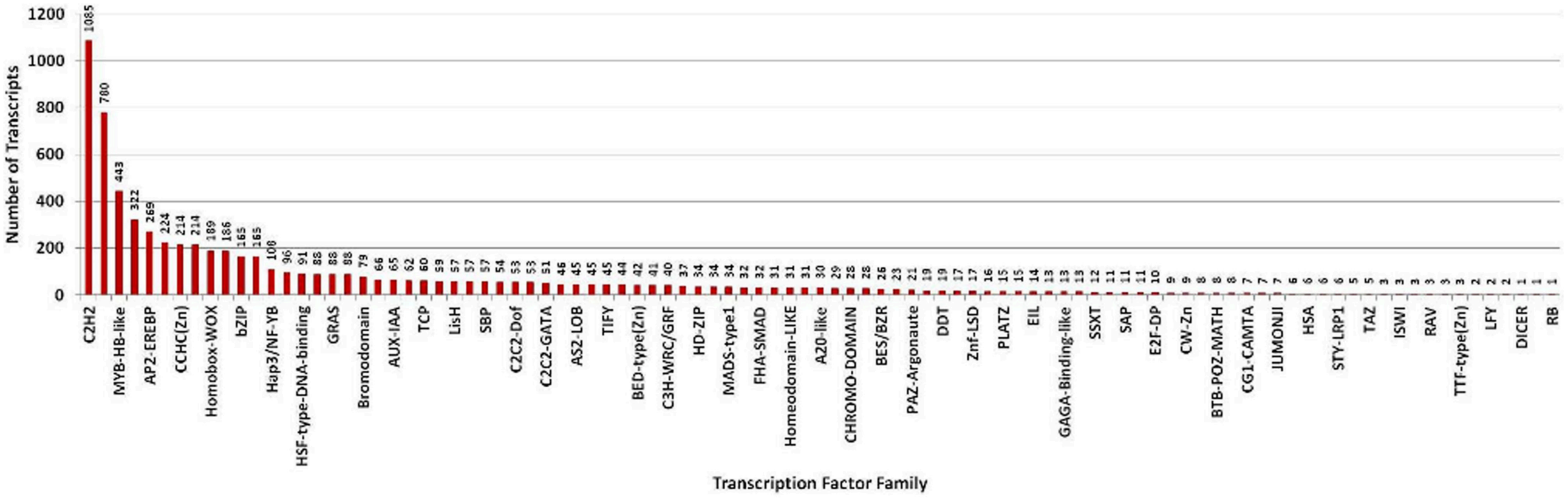
**Distribution of transcripts annotated to both plant-specific and plant-non-specific transcription factor families**.

**Figure 8:**
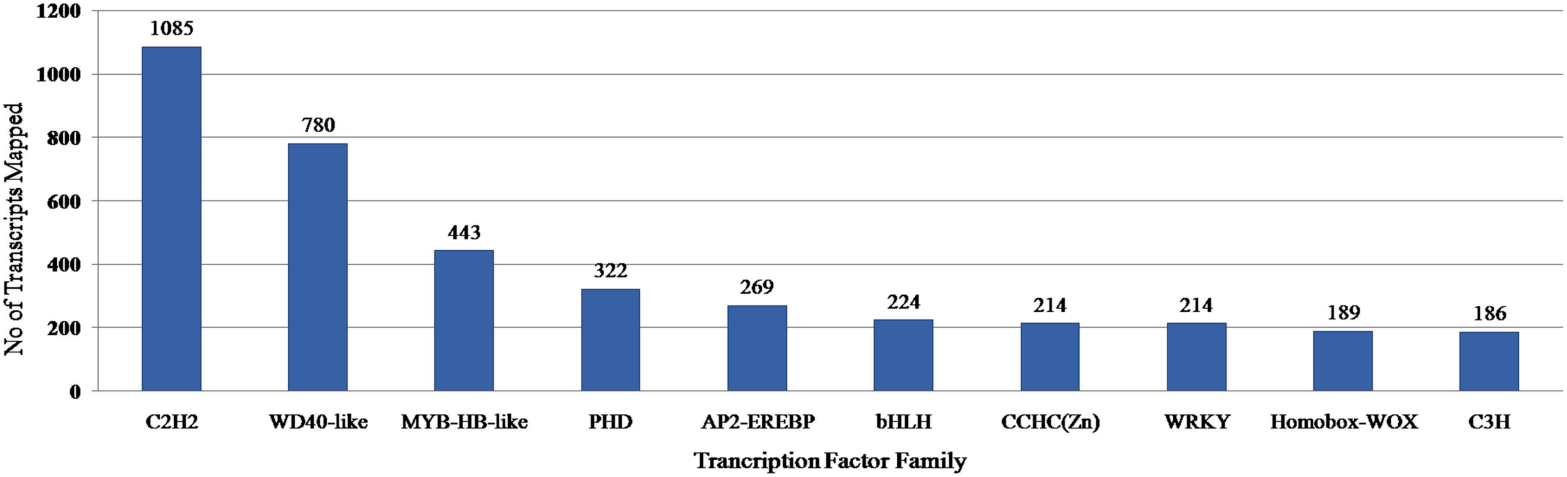
**PlantTFcat analysis of ***A. paniculata*** transcriptome showing distribution of transcription factors in top 10 families**.

Transcription factors regulate the gene expression patterns which in turn determine various biological processes. Out of 83,800 clustered unigene transcripts, 6767 unique transcripts belonged to 97 plant-specific and plant-non-specific transcription factor families (Figure 7). Among the identified unigenes, 30.93% belonged to the category of plant-specific transcription factor family. Most of them represent AP2EREBP family followed by WRKY, NAM, B3-Domain etc. While 69.07% of the unigenes were grouped into plant non-specific transcription factor family and were classified into families such as C2H2, WD40-like, MYB-HB-like etc (Figure 8). A total of 25 different functional domains were identified. Of the 6767 TF coding transcripts, 2498 transcripts belonged to the DNA-binding domain, while 147 transcripts represented different InterPro domains.

### Classification of cytochrome P450s expressed in leaf

**Figure 9:**
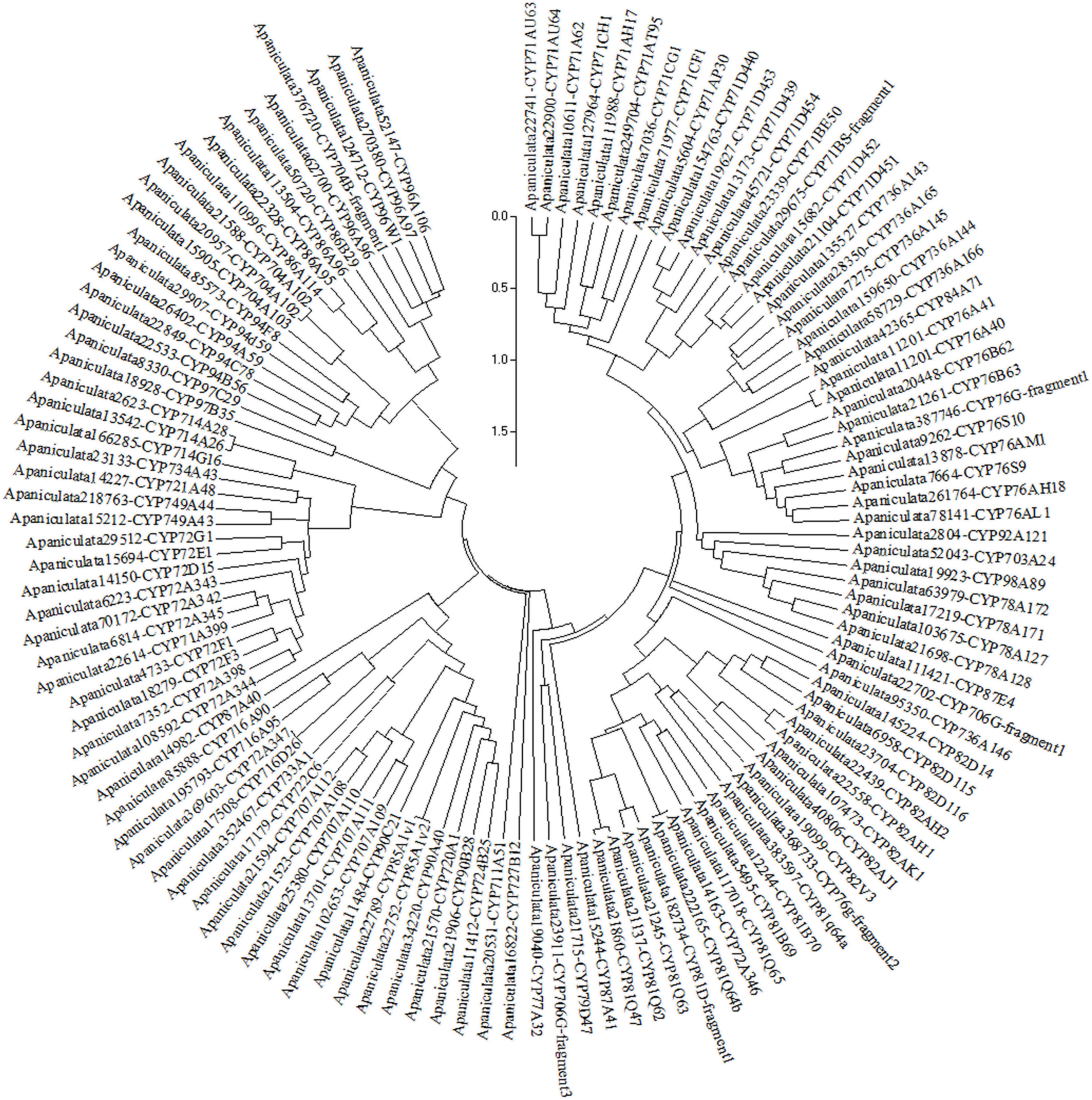
**Phylogenetic tree of cytochrome P450 proteins expressed in the leaf of ***A. paniculata*** (tree was built with bootstrap testing using 1000 replications)**.

Deduced proteome of *A. paniculata* leaf transcriptome revealed expression of 124 CYP450 transcripts belonging to seven clans such as CYP71, CYP72, CYP85, CYP86, CYP97, CYP711, and CYP727. A maximum of 64 CYP transcripts expressed were from the clan CYP71 representing families viz., CYP71, CYP76, CYP78, CYP79, CYP81, CYP82, CYP84, CYP92, CYP98, CYP703, CYP706, and CYP736. Nineteen CYP transcripts expressed were from the clan CYP85 representing the families CYP85, CYP87, CYP90, CYP707, CYP716, CYP720, CYP722, CYP724, and CYP733. A total of 20 transcripts were expressed from families CYP72, CYP714, CYP721, CYP734, and CYP749 pertaining to the clan CYP72. From CYP86 clan 17 expressed transcripts were classified into four families, CYP86, CYP94, CYP96, and CYP704. Single family clans, viz., CYP97, CYP711, and CYP727 are represented by the expression of two, one and one gene, respectively, (Figure 9) (Supplementary File S7).

**Figure 10:**
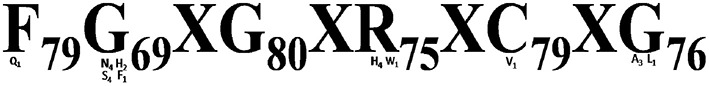
**Conserved heme motif of deduced CYP450s expressed in the leaf of ***A. paniculata*****.

All the 124 deduced CYP450 proteins contained 66 to 584 amino acid residues. Majority (96) of these proteins contained 301 to 584 amino acid residues followed by 15 proteins with 200–300 amino acid residues, while the remaining 13 proteins contained 66–99 amino acid residues. CYP450s expressed in the *A. paniculata* leaf displayed a wide range (5.11 to 11.6) of iso-electric point (pI) (Supplementary File S8), and the conserved signature motif of F_79_G_69_XG_80_XR_75_XC_79_XG_76_ (Figure 10).

### Identification of SSRs

SSRs also known as microsatellites, are short repeating sequences with a unit size of mono-, di-, tri-, tetra-, or penta-nucleotides. MISA analysis of 83,800 clustered transcripts, revealed a total number of 32,341 SSRs in 23,168 transcripts (Supplementary File S9). More than 1 SSR was found in 6540 transcripts including 2438 transcripts with compound SSRs. A maximum number (12,094) of SSRs were identified as di-nucleotide repeats followed by tri-nucleotide (9599), mono-nucleotide (9139), tetra-nucleotide (1377), and penta-nucloetide (132) repeats.

### Expression levels of selected transcripts by qRT-PCR

**Figure 11:**
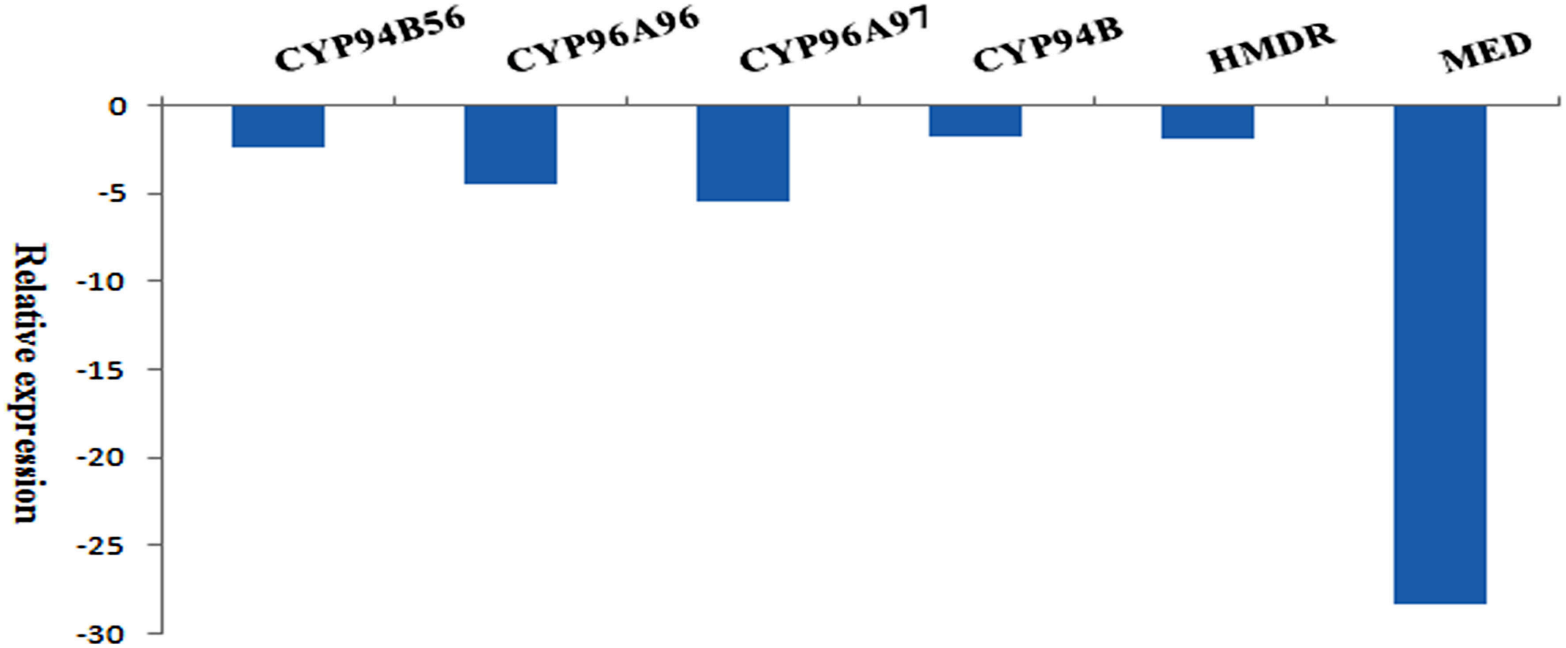
**qRT-PCR of selected transcripts and their relative expression levels with reference to Actin (Apaniculata22671, len = 1798; control): CYP94B56 (Apaniculata22533, len = 6227), CYP96A96 (Apaniculata62700, len = 1891), CYP96A97 (Apaniculata270380, len = 675), CYP94B (Apaniculata474149, len = 415), 4-hydroxy-3-methylbut-2-enyl diphosphate reductase (HMDR; Apaniculata23282, len = 1733), 2-C-methyl-D-erythritol 4-phosphate cytidylyltransferase (MED; Apaniculata24573, len = 1282)**.

To validate the authenticity of assembled transcripts, expression of the selected transcripts (House-keeping actin, CYP94B56, CYP96A96, CYP96A97, and CYP94B), and two transcripts coding for enzymes involved in terpenoid biosynthesis such as 4-hydroxy-3-methylbut-2-enyl diphosphate reductase (HMDR), and 2-C-methyl-D-erythritol 4-phosphate cytidylyltransferase (MED) were used for qRT-PCR analysis. The relative fold expression of each transcript with actin was given in Figure 11. When compared to actin, CYP94B showed −1.7423 fold decreased expression level followed by HMDR (−1.8015), CYP94B56 (−2.3311), CYP96A96 (−4.6055), CYP96A97 (−5.4189), and MED (−28.2857).

## Discussion

Trancriptome sequencing is a high-throughput and cost effective method for generating genomic resources in non-model organisms sans genomic information. Application of NGS technology in the characterization of transcriptome of non-model species is well-documented. Illumina HiSeq™ 2000 sequencing is known to generate sizable data with 98% accuracy, which in turn can be used for the efficient *de novo* assembly of transcriptome. *A. paniculata* leaf transcriptome analysis has been carried out as leaf is the primary site of synthesis and accumulation of bioactive andrographolide. This investigation, employing Illumina platform, recorded an N50 of 1880 bp which is much higher compared to N50 values (290–1515 bp) reported from the previous studies with non-model plants such as *Carthamus tinctorious* (Lulin et al., 2012), *C. longa* (Annadurai et al., 2013), *Cocos nucifera* (Fan et al., 2013), *Raphanus sativus* (Zhang et al., 2013), *Centella asiatica* (Sangwan et al., 2013), and *A. paniculata* (Garg et al., 2015), thereby indicating the better *de novo* assembly of transcriptome. Remaining 35.4% transcripts were uncharacterized owing to their uniqueness or incompleteness of the current database. However, blast results showed nearly 64.6% significant hits against plant NRDB and CDD supporting the validity of *de novo* assembly of the transcriptome. The present investigation on *A. paniculata* transcriptome revealed that majority of assembled transcripts with long ORFs coding for functional proteins, which may be attributed to the effectiveness of trinity tool, sequence depth and homozygous nature of the plant material. An overall annotation (64.6%) using plant NRDB and CDD proved better than the previous transcriptome studies on *Carthamus tinctorious* (58%), *C. longa* (54.6%), and *Centella asiatica* (53.04%).

Cytochrome P450s (CYPs) belonging to super family proteins representing one percent of the proteome in plants were found to mediate oxidation of various substrates involved in both primary and secondary metabolism (Babu et al., 2013; Kumar et al., 2014; Mittapelli et al., 2014). Transcriptome analysis revealed a total of 124 CYP450 transcripts expressed in the leaf tissue of *A. paniculata*, representing 34 families belonging to 7 clans. Among these clans, CYP71 contained a maximum number of 64 CYP450 transcripts. It was reported that, 116 full length CYP450 transcripts were identified in *Salvia miltorrhiza* (Chen et al., 2014). Recently, 52 CYP450s were identified to play crucial roles in the terpenoid biosynthesis in various medicinal plants and majority (18) of them belonged to CYP71 family (Zhao et al., 2014).

Out of 124 assembled transcripts encoding CYP450s, 114 sequences represent functional proteins. Further, proper assembly of alternatively spliced transcripts for CYP704A2 and transcripts of duplicate genes encoding for CYP85A1v1 and CYPA1v2 clearly demonstrate the effective assembly of transcriptome in the non-model plant *A. paniculata*. Analysis of CYP450s representing 1% of the proteome might be useful in assessing the quality of assembled transcriptome.

A large number (14,720) of unique KOG mapped transcripts and 71 unique domains identified in the transcriptome provide greater insight into the gene content, biological processes, and pathways conserved in *A. paniculata*. Identification of candidate genes and key enzymes is crucial in understanding the biosynthetic pathways of functional isoprenoids in *A. paniculata*. As pharmaceutical properties of *A. paniculata* largely depend on its terpenoid profile, the present study was mainly focused on the identification of transcripts involved in isoprenoid biosynthesis. The KEGG predictions of the present study mapped 216 transcripts encoding for various enzymes involved in the biosynthesis of different isoprenoids such as mono-terpenes, di-terpenes, tri-terpenes, and ubiquinones. A large number of assembled transcripts coding for proteins with more than 300 aa indicates the potential of high-throughput sequencing for identifying transcripts corresponding to various metabolic pathways including that of andrographolide biosynthesis.

In the leaf of *A. paniculata*, andrographolides are synthesized using MVA and MEP/DXP pathways. Overall 35 terpene synthases identified were classified into different groups. Enzymes exhibiting class I activity contained the motif DDXXD responsible for metal-dependent ionization of prenyl-diphosphate substrates. Other enzymes having DXDD motif specify class II enzymatic activity required for circularization of GGPP. Third group of terpene synthases mainly contained EDXXD motif responsible for class II diterpene synthase activity. Identification of transcripts coding for 3-hydroxy-3-methylglutaryl coenzyme A synthase (HMGR), in the present investigation, corroborates with an earlier study demonstrating this enzyme as one of the key players in the accumulation of andrographolide (Jha et al., 2011). The formation of cyclic diterpenes can be streamlined by taking into account the different types of cyclization of geranyl geranyl diphosphate (GGPP). It was demonstrated that the involvement of Type-B cyclization is necessary for the biosynthesis of labdanes and ent-labdane. Also, it was suggested that labdanes are derived from GGPP by a two step process via mono-cyclic intermediates. In a previous study, the formation of *ent*-CPP from GGPP was observed in tobacco (Guo et al., 1994). Transcripts coding for key enzymes like geranyl geranyl pyrophosphate synthase, ent-copalyl diphosphate synthase (*ent*-CPP), phosphomevalonate kinase-like, etc identified in the present study, are useful to elucidate the processes involved in diterpene biosynthesis.

In this study, for the identification of transcription factors, PlantTFcat, an online tool was used as it not only categorized plant TF/TR/CR separately but also provided accurate results with lesser false positives than BLAST based programmes. Out of 6767 transcripts identified, 5083 were mapped to plant TFs while the remaining transcripts were mapped to various regulators of transcription, chromatin structure/its remodeling and transcriptional activation families, suggesting the efficacy of PlantTFcat tool for identification of various proteins involved in the regulation of transcription. In this investigation, di- and tri-nucleotide repeats were found more frequently than tetra- and penta-nucleotide repeats, as observed in earlier studies (Toledo-Silva et al., 2013; Torales et al., 2013). However, frequency and distribution of SSRs primarily depend on the employed dataset, tools, and criteria used. Earlier, it was found that gene regulation, transcription and protein function typically depend on the number of repeating units (Kashi and King, 2006). The SSRs resource developed in this study will be of great use in assessing the genetic diversity and identification of genotypes of *A. paniculata* having desirable terpenoid profiles.

Earlier report on *A. paniculata* transcriptome indicated the synthesis of bioactive andrographolide takes place primarily in the leaf (Garg et al., 2015). The present study generated additional transcripts as evidenced by greater number (83,800) of leaf transcripts as compared to earlier study (69,011). Further, N50 of 1880 bp is much higher than 926 bp of earlier study indicating the greater depth of sequence. Present study also annotated 49,363 transcripts which is much higher compared to 40,586 annotated leaf transcripts of earlier report indicating the possible complementary nature of the data besides additional transcripts useful for further studies. Details pertaining to the advantages of the present investigation are presented in Supplementary File S10.

Transcript information generated in the present study has great relevance in functional genomics of non-model plant *A. paniculata* for elucidating isoprenoid biosynthetic pathway. Further, the information can also be used for overexpressing certain genes for enhanced production of bioactive diterpenoids. Generated SSR information is useful in fingerprinting of the germplasm and screening for different ecotypes. The transcription factors identified can be used for the development of pest resistant/ abiotic stress tolerant transgenics in other plants of economic importance. Variant cytochrome P450s identified in this study will facilitate novel cyclization reactions and helpful in the synthesis of designer molecules.

To sum up, in this investigation, an attempt has been made to characterize the leaf transcriptome of *A. paniculata*, which helps in the identification of various transcripts involved in the synthesis of secondary metabolites. The assembled sequence information generated herein is made available for the first time in the TSA database which serves as a valuable genomic resource. The identified transcripts of terpenoid biosynthesis will be useful for further genetic analysis of andrographolide biosyntheses and their pathway engineering.

## Author contributions

DV and VK conceived and designed the experimental plan. NC, MD, and SM performed experiments. DV and VK analyzed and interpreted sequence data and drafting of the manuscript. All authors read and approved the final manuscript.

### Conflict of interest statement

The authors declare that the research was conducted in the absence of any commercial or financial relationships that could be construed as a potential conflict of interest.

## Supplementary material

Supplementary Figure 1 **Functional distribution of KOG annotated transcripts classifying into 25 categories**.

Supplementary File S1 **List of primers designed from annotated gene sequences of ***A. paniculata*** transcriptome for qRT-PCR validation**.

Supplementary File S2 **Annotated transcripts mapped to various CDD domains with an ***E***-value cutoff of 0.01**.

Supplementary File S3 **Overview of the leaf transcriptome of ***A. paniculata*** (A) Transcript annotation (B) Biological process (C) Cellular components (D) Molecular functions of annotated transcripts**.

Supplementary File S4 **Description of KOG mapped transcripts with their corresponding enzyme codes**.

Supplementary File S5 **Transcripts annotated in the KEGG pathway**.

Supplementary File S6 **Properties of enzymes involved in terpenoid biosynthesis from ***A. paniculata*** leaf transcriptome**.

Supplementary File S7 **Family wise distribution of cytochrome P450 transcripts expressed in the leaf of ***A. paniculata*****.

Supplementary File S8 **Classification and properties of deduced Cytochrome P450s in ***A. paniculata*** leaf transcriptome**.

Supplementary File S9 **Summary of simple sequence repeats identified in annotated transcripts**.

Supplementary File S10 **Advantages of present transcriptome analysis over the earlier work published**.
