# Supplementary figures and images for: De novo Assembly of Leaf Transcriptome in the Medicinal Plant Andrographis paniculata
Source: Front Plant Sci. 2016 Aug 17;7:1203. doi: 10.3389/fpls.2016.01203 (PMC4987368; doi:10.3389/fpls.2016.01203)

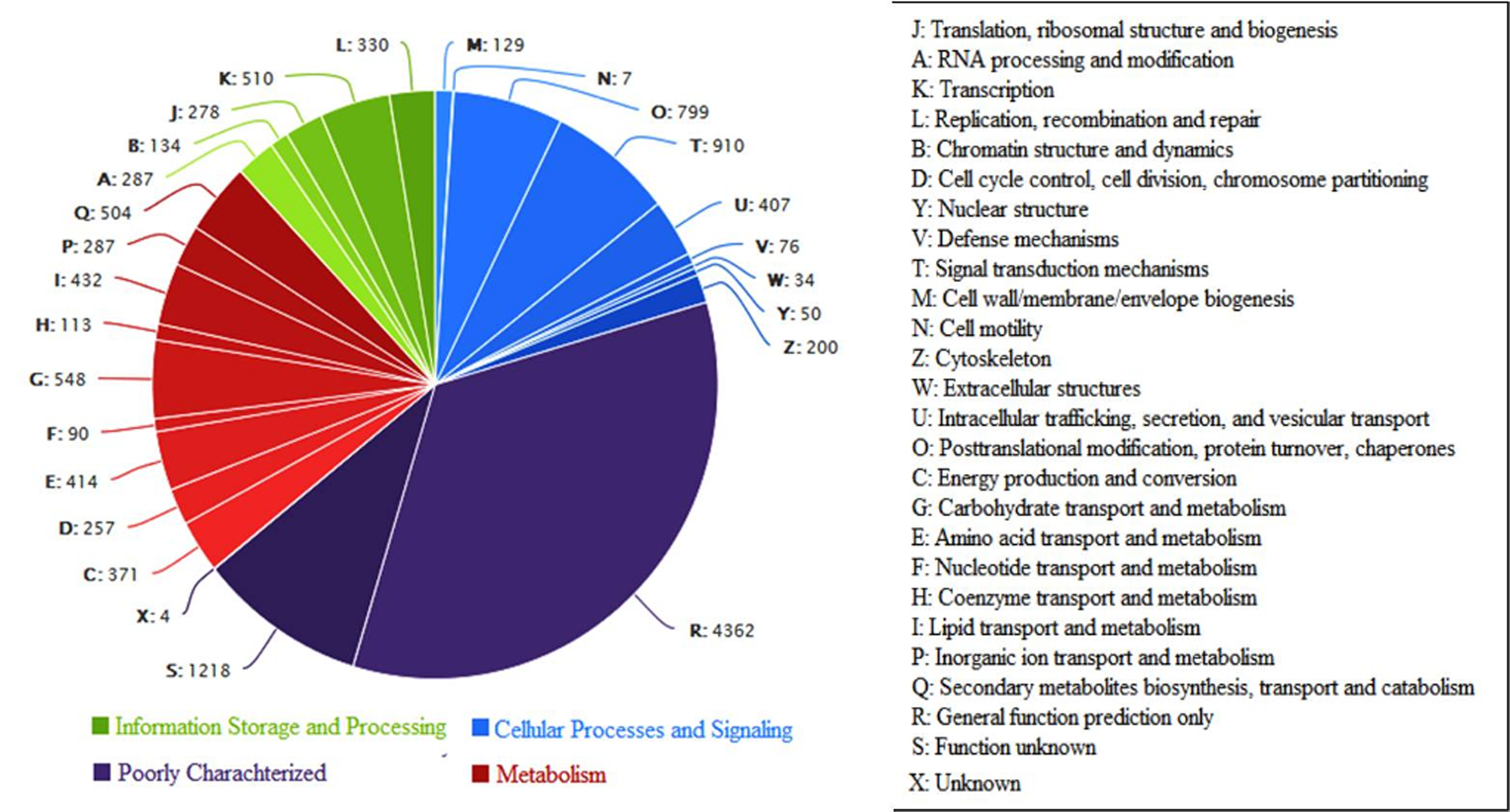

Supplement: Supplementary Figure 1 — Functional distribution of KOG annotated transcripts classifying into 25 categories. [file Image1.TIF]
