# Supplementary material for: De novo Assembly of Leaf Transcriptome in the Medicinal Plant Andrographis paniculata
Source: Front Plant Sci. 2016 Aug 17;7:1203. doi: 10.3389/fpls.2016.01203 (PMC4987368; doi:10.3389/fpls.2016.01203)
Supplement: Supplementary File S1 — List of primers designed from annotated gene sequences of A. paniculata transcriptome for qRT-PCR validation. [file Table1.DOCX]

| 1 | CY94B56F | TTGTGGAGGAGGTGAGCT |
| --- | --- | --- |
| 2 | CY94B56R | AATACGTCACCCGGTTGC |
| 3 | CY96A96F | CTAACAAGAAGTGGCATGTCAT |
| 4 | CY96A96R | CCCATATCGACTCCATCCTC |
| 5 | CY96A97F | GCCAGACTATGTCGTTCCTG |
| 6 | CY96A97R | GCCTTCTCGCTCGGCACATT |
| 7 | CYP94BF | AGGAGAGGCGGTTATGAGG |
| 8 | CYP94BR | GACTTGAGCCACGTCATCCCT |
| 9 | HMDRF | CTATTTGTGACGCAACTCAAG |
| 10 | HMDRR | CCAACTGTGACCGGACCCTT |
| 11 | MEDF | TCTGAGCTTGTGTGCATCCAT |
| 12 | MEDR | CTCTATTGACAAGCTCGAATCC |
| 13 | ActinF | AGGGACATCAAAGAGAAACTG |
| 14 | ActinR | GCTGTGATCTCCTTGCTCATT |

**S1 Table.** List of primers designed from annotated gene sequences of *A. paniculata* transcriptome for qRT-PCR validation
