# Supplementary material for: De novo Assembly of Leaf Transcriptome in the Medicinal Plant Andrographis paniculata
Source: Front Plant Sci. 2016 Aug 17;7:1203. doi: 10.3389/fpls.2016.01203 (PMC4987368; doi:10.3389/fpls.2016.01203)
Supplement: Supplementary File S2 — Annotated transcripts mapped to various CDD domains with an E-value cutoff of 0.01. [file Table2.DOCX]

| **Domain_ID** | **Domain_Name** | **No Transcripts Mapped** | **CDD_ID** | **Gene_Name** |
| --- | --- | --- | --- | --- |
| PRK12270 | alpha-ketoglutarate decarboxylase; Reviewed | 975 | gnl\|CDD\|237030 | Kg0d |
| COG3889 | Predicted solute binding protein [General function prediction only] | 298 | gnl\|CDD\|226406 | COG3889 |
| pfam11593 | Mediator complex subunit 3 fungal. Mediator is a large complex of up to 33 proteins that is conserved from plants to fungi to humans - the number and representation of individual subunits varying with species. It is arranged into four different sections | 278 | gnl\|CDD\|221143 | Med3 |
| PTZ00436 | 60S ribosomal protein L19-like protein; Provisional | 136 | gnl\|CDD\|185616 | PTZ00436 |
| PRK02888 | nitrous-oxide reductase; Validated | 86 | gnl\|CDD\|235082 | PRK02888 |
| PRK07003 | DNA polymerase III subunits gamma and tau; Validated | 45 | gnl\|CDD\|235906 | PRK07003 |
| PRK12678 | transcription termination factor Rho; Provisional | 25 | gnl\|CDD\|237171 | PRK12678 |
| PRK11907 | bifunctional 2' | 13 | gnl\|CDD\|237019 | PRK11907 |
| PRK07735 | NADH dehydrogenase subunit C; Validated | 12 | gnl\|CDD\|236081 | PRK07735 |
| PRK09510 | cell envelope integrity inner membrane protein TolA; Provisional | 6 | gnl\|CDD\|236545 | tolA |
| PRK14724 | DNA topoisomerase III; Provisional | 5 | gnl\|CDD\|237803 | PRK14724 |
| TIGR04244 | nitrous-oxide reductase | 5 | gnl\|CDD\|234519 | nitrous_NosZ_RR |
| PRK14293 | chaperone protein DnaJ; Provisional | 4 | gnl\|CDD\|237663 | PRK14293 |
| PRK14298 | chaperone protein DnaJ; Provisional | 2 | gnl\|CDD\|184612 | PRK14298 |
| PTZ00046 | rifin; Provisional | 2 | gnl\|CDD\|240242 | PTZ00046 |
| TIGR03734 | PRTRC system ParB family protein. A novel genetic system characterized by six major proteins | 1 | gnl\|CDD\|234336 | PRTRC_parB |

**Supplementary File S2.** Annotated transcripts mapped to various CDD domains with an E-value cutoff of 0.01
