# Supplementary material for: De novo Assembly of Leaf Transcriptome in the Medicinal Plant Andrographis paniculata
Source: Front Plant Sci. 2016 Aug 17;7:1203. doi: 10.3389/fpls.2016.01203 (PMC4987368; doi:10.3389/fpls.2016.01203)
Supplement: Supplementary File S5 — Transcripts annotated in the KEGG pathway. [file Table5.docx]

| **Supplementary File S5. Transcripts annotated in the KEGG pathway** | | |
| --- | --- | --- |
| **Pathway ID** | **Pathway** | **Sequences in Pathway** |
| map00500 | Starch and sucrose metabolism | 666 |
| map00230 | Purine metabolism | 639 |
| map00240 | Pyrimidine metabolism | 297 |
| map00040 | Pentose and glucuronate interconversions | 284 |
| map00010 | Glycolysis / Gluconeogenesis | 279 |
| map00564 | Glycerophospholipid metabolism | 262 |
| map00561 | Glycerolipid metabolism | 258 |
| map00052 | Galactose metabolism | 248 |
| map04660 | T cell receptor signaling pathway | 245 |
| map00520 | Amino sugar and nucleotide sugar metabolism | 239 |
| map00360 | Phenylalanine metabolism | 217 |
| map00620 | Pyruvate metabolism | 214 |
| map04070 | Phosphatidylinositol signaling system | 195 |
| map00330 | Arginine and proline metabolism | 182 |
| map00940 | Phenylpropanoid biosynthesis | 177 |
| map00627 | Aminobenzoate degradation | 175 |
| map00270 | Cysteine and methionine metabolism | 174 |
| map00190 | Oxidative phosphorylation | 168 |
| map00710 | Carbon fixation in photosynthetic organisms | 161 |
| map00630 | Glyoxylate and dicarboxylate metabolism | 154 |
| map00071 | Fatty acid degradation | 146 |
| map00680 | Methane metabolism | 145 |
| map00260 | Glycine, serine and threonine metabolism | 143 |
| map00600 | Sphingolipid metabolism | 140 |
| map00030 | Pentose phosphate pathway | 139 |
| map00051 | Fructose and mannose metabolism | 138 |
| map00380 | Tryptophan metabolism | 136 |
| map00280 | Valine, leucine and isoleucine degradation | 132 |
| map00740 | Riboflavin metabolism | 129 |
| map00250 | Alanine, aspartate and glutamate metabolism | 126 |
| map00562 | Inositol phosphate metabolism | 126 |
| map00480 | Glutathione metabolism | 125 |
| map00982 | Drug metabolism - cytochrome P450 | 124 |
| map00760 | Nicotinate and nicotinamide metabolism | 120 |
| map00350 | Tyrosine metabolism | 119 |
| map00592 | alpha-Linolenic acid metabolism | 117 |
| map00730 | Thiamine metabolism | 117 |
| map00511 | Other glycan degradation | 109 |
| map00310 | Lysine degradation | 103 |
| map00720 | Carbon fixation pathways in prokaryotes | 101 |
| map00983 | Drug metabolism - other enzymes | 100 |
| map00860 | Porphyrin and chlorophyll metabolism | 100 |
| map00020 | Citrate cycle (TCA cycle) | 99 |
| map00980 | Metabolism of xenobiotics by cytochrome P450 | 97 |
| map00900 | Terpenoid backbone biosynthesis | 96 |
| map00410 | beta-Alanine metabolism | 94 |
| map00053 | Ascorbate and aldarate metabolism | 93 |
| map00970 | Aminoacyl-tRNA biosynthesis | 89 |
| map00565 | Ether lipid metabolism | 89 |
| map04150 | mTOR signaling pathway | 89 |
| map00340 | Histidine metabolism | 86 |
| map00061 | Fatty acid biosynthesis | 84 |
| map00400 | Phenylalanine, tyrosine and tryptophan biosynthesis | 84 |
| map00830 | Retinol metabolism | 82 |
| map00450 | Selenocompound metabolism | 75 |
| map00531 | Glycosaminoglycan degradation | 74 |
| map00770 | Pantothenate and CoA biosynthesis | 73 |
| map00604 | Glycosphingolipid biosynthesis - ganglio series | 72 |
| map00910 | Nitrogen metabolism | 70 |
| map00625 | Chloroalkane and chloroalkene degradation | 66 |
| map00920 | Sulfur metabolism | 64 |
| map01040 | Biosynthesis of unsaturated fatty acids | 61 |
| map00591 | Linoleic acid metabolism | 60 |
| map00950 | Isoquinoline alkaloid biosynthesis | 59 |
| map00640 | Propanoate metabolism | 59 |
| map00130 | Ubiquinone and other terpenoid-quinone biosynthesis | 59 |
| map00590 | Arachidonic acid metabolism | 58 |
| map00780 | Biotin metabolism | 58 |
| map00650 | Butanoate metabolism | 58 |
| map00906 | Carotenoid biosynthesis | 58 |
| map00670 | One carbon pool by folate | 58 |
| map00790 | Folate biosynthesis | 56 |
| map00510 | N-Glycan biosynthesis | 54 |
| map00513 | Various types of N-glycan biosynthesis | 54 |
| map00941 | Flavonoid biosynthesis | 53 |
| map00960 | Tropane, piperidine and pyridine alkaloid biosynthesis | 53 |
| map00290 | Valine, leucine and isoleucine biosynthesis | 53 |
| map00300 | Lysine biosynthesis | 51 |
| map00460 | Cyanoamino acid metabolism | 49 |
| map00603 | Glycosphingolipid biosynthesis - globo series | 47 |
| map00140 | Steroid hormone biosynthesis | 45 |
| map00534 | Glycosaminoglycan biosynthesis - heparan sulfate / heparin | 42 |
| map00903 | Limonene and pinene degradation | 36 |
| map00966 | Glucosinolate biosynthesis | 35 |
| map00401 | Novobiocin biosynthesis | 33 |
| map00626 | Naphthalene degradation | 32 |
| map00100 | Steroid biosynthesis | 32 |
| map00521 | Streptomycin biosynthesis | 29 |
| map00232 | Caffeine metabolism | 28 |
| map00281 | Geraniol degradation | 28 |
| map00062 | Fatty acid elongation | 27 |
| map00901 | Indole alkaloid biosynthesis | 27 |
| map00750 | Vitamin B6 metabolism | 25 |
| map00332 | Carbapenem biosynthesis | 24 |
| map00904 | Diterpenoid biosynthesis | 24 |
| map00660 | C5-Branched dibasic acid metabolism | 22 |
| map00073 | Cutin, suberine and wax biosynthesis | 22 |
| map00532 | Glycosaminoglycan biosynthesis - chondroitin sulfate / dermatan sulfate | 22 |
| map00430 | Taurine and hypotaurine metabolism | 22 |
| map00902 | Monoterpenoid biosynthesis | 21 |
| map00908 | Zeatin biosynthesis | 21 |
| map00261 | Monobactam biosynthesis | 19 |
| map00930 | Caprolactam degradation | 18 |
| map00120 | Primary bile acid biosynthesis | 18 |
| map00984 | Steroid degradation | 17 |
| map00072 | Synthesis and degradation of ketone bodies | 15 |
| map00362 | Benzoate degradation | 14 |
| map00524 | Butirosin and neomycin biosynthesis | 13 |
| map00944 | Flavone and flavonol biosynthesis | 13 |
| map00909 | Sesquiterpenoid and triterpenoid biosynthesis | 13 |
| map00254 | Aflatoxin biosynthesis | 12 |
| map00440 | Phosphonate and phosphinate metabolism | 12 |
| map00253 | Tetracycline biosynthesis | 12 |
| map00791 | Atrazine degradation | 11 |
| map00471 | D-Glutamine and D-glutamate metabolism | 11 |
| map00945 | Stilbenoid, diarylheptanoid and gingerol biosynthesis | 11 |
| map00540 | Lipopolysaccharide biosynthesis | 10 |
| map00550 | Peptidoglycan biosynthesis | 10 |
| map00643 | Styrene degradation | 9 |
| map00623 | Toluene degradation | 9 |
| map00361 | Chlorocyclohexane and chlorobenzene degradation | 8 |
| map00981 | Insect hormone biosynthesis | 8 |
| map00942 | Anthocyanin biosynthesis | 7 |
| map00514 | Other types of O-glycan biosynthesis | 7 |
| map00473 | D-Alanine metabolism | 6 |
| map00943 | Isoflavonoid biosynthesis | 6 |
| map00785 | Lipoic acid metabolism | 6 |
| map00523 | Polyketide sugar unit biosynthesis | 6 |
| map01051 | Biosynthesis of ansamycins | 5 |
| map01055 | Biosynthesis of vancomycin group antibiotics | 4 |
| map00642 | Ethylbenzene degradation | 4 |
| map00563 | Glycosylphosphatidylinositol(GPI)-anchor biosynthesis | 4 |
| map01053 | Biosynthesis of siderophore group nonribosomal peptides | 3 |
| map01062 | Biosynthesis of terpenoids and steroids | 3 |
| map00311 | Penicillin and cephalosporin biosynthesis | 3 |
| map00195 | Photosynthesis | 3 |
| map00402 | Benzoxazinoid biosynthesis | 2 |
| map00965 | Betalain biosynthesis | 2 |
| map00905 | Brassinosteroid biosynthesis | 2 |
| map00472 | D-Arginine and D-ornithine metabolism | 1 |
