# Supplementary material for: De novo Assembly of Leaf Transcriptome in the Medicinal Plant Andrographis paniculata
Source: Front Plant Sci. 2016 Aug 17;7:1203. doi: 10.3389/fpls.2016.01203 (PMC4987368; doi:10.3389/fpls.2016.01203)
Supplement: Supplementary File S6 — Properties of enzymes involved in terpenoid biosynthesis from A. paniculata leaf transcriptome. [file Table6.docx]

| **Supplementary File S6. Properties of enzymes involved in terpenoid biosynthesis from *A.paniculata* leaf transcriptome** | | | | | | | | | | | | |  |
| --- | --- | --- | --- | --- | --- | --- | --- | --- | --- | --- | --- | --- | --- |
| **Transcript ID** | **Protein Length (amino acids)** | **pI** | **MW (kD)** | | **Ezyme ID** | | **Enzyme Name** | | **Relative Expression**  **(RPKM)** | | **Terpene synthase Motif** | |  |
| Apaniculata15136_c0_seq1_len=1338 | 304 | 5.7 | 33.1 | | ec:1.1.1.2 | | alcohol dehydrogenase (NADP+) | | -2.98 | |  | |  |
| Apaniculata22271_c0_seq18_len=863 | 113 | 10.51 | 13.28 | | ec:1.1.1.2 | | dehydrogenase (NADP+) | | -6.57 | |  | |  |
| Apaniculata22271_c0_seq26_len=599 | 163 | 8.73 | 17.53 | | ec:1.1.1.2 | | (+)-neomenthol dehydrogenase (NADP+) | | -4.86 | |  | |  |
| Apaniculata9975_c0_seq1_len=713 | 213 | 6.5 | 23.16 | | ec:1.1.1.2 | | (+)-neomenthol dehydrogenase (NADP+) | | -6.1 | |  | |  |
| Apaniculata123237_c0_seq1_len=749 | 225 | 6.18 | 25.29 | | ec:1.1.1.216 | | reductase | | -7.3 | |  | |  |
| Apaniculata13389_c0_seq2_len=1310 | 344 | 7.7 | 37.37 | | ec:1.1.1.216 | | reductase | | -3.65 | |  | |  |
| Apaniculata13389_c0_seq2_len=1310 | 344 | 7.7 | 37.37 | | ec:1.1.1.216 | | dehydrogenase (NADP+) | | -3.65 | |  | |  |
| Apaniculata11747_c0_seq1_len=1878 | 328 | 5.96 | 36.21 | | ec:1.1.1.237 | | hydroxyphenylpyruvate reductase | | -3.27 | |  | |  |
| Apaniculata63701_c0_seq1_len=1242 | 329 | 5.52 | 36.22 | | ec:1.1.1.237 | | hydroxyphenylpyruvate reductase | | -4.73 | | DDVAD | |  |
| Apaniculata12511_c0_seq1_len=1103 | 315 | 6.06 | 33.03 | | ec:1.1.1.243 | | (-)-isopiperitenol/(-)-carveol dehydrogenase | | 1.88 | |  | |  |
| Apaniculata8715_c0_seq1_len=840 | 259 | 5.09 | 27.03 | | ec:1.1.1.243 | | (-)-isopiperitenol/(-)-carveol dehydrogenase | | -4.03 | | DIQDD | |  |
| Apaniculata23665_c0_seq1_len=1918 | 543 | 6.24 | 59.25 | | ec:1.1.1.267 | | 1-deoxy-D-xylulose 5-phosphate reductoisomerase, chloroplastic | | 1.17 | |  | |  |
| Apaniculata24516_c0_seq1_len=870 | 245 | 5.17 | 27.16 | | ec:1.1.1.267 | | 1-deoxy-D-xylulose-5-phosphate reductoisomerase | | 0.13 | |  | |  |
| Apaniculata25393_c0_seq1_len=835 | 219 | 9.26 | 23.14 | | ec:1.1.1.267 | | 1-deoxy-D-xylulose-5-phosphate reductoisomerase | | -0.58 | |  | |  |
| Apaniculata21538_c0_seq4_len=2369 | 665 | 6.31 | 71.32 | | ec:1.1.1.34 | | 3-hydroxy-3-methylglutaryl-coenzyme A reductase (NADPH) | | -1.53 | | DDLLD | |  |
| Apaniculata10571_c0_seq1_len=1639 | 546 | 7.5 | 58.81 | | ec:1.1.1.88 | | 3-hydroxy-3-methylglutaryl-coenzyme A reductase 1 | | -2.69 | |  | |  |
| Apaniculata24030_c0_seq1_len=1768 | 497 | 5.83 | 54.74 | | ec:1.13.11.27 | | hydroxyphenylpyruvate dioxygenase | | 0.65 | | DRDD | |  |
| Apaniculata22488_c1_seq2_len=623 | 107 | 9.99 | 12.33 | | ec:1.14.11.13 | | 2beta-dioxygenase | | -3.7 | |  | |  |
| Apaniculata22187_c0_seq13_len=1449 | 149 | 4.95 | 16.67 | | ec:1.14.11.13 | | 2beta-dioxygenase | | -4.07 | |  | |  |
| Apaniculata387173_c0_seq1_len=335 | 111 | 7.88 | 12.01 | | ec:1.14.11.15 | | 3beta-dioxygenase | | -7.64 | |  | |  |
| Apaniculata462388_c0_seq1_len=291 | 97 | 5.46 | 10.73 | | ec:1.14.11.15 | | 3beta-dioxygenase (2-oxoglutarate-dependent) | | -7.3 | |  | |  |
| Apaniculata470987_c0_seq1_len=244 | 81 | 6.9 | 9.27 | | ec:1.14.11.15 | | 3beta-dioxygenase | | -7.75 | |  | |  |
| Apaniculata57539_c0_seq1_len=1204 | 354 | 5.77 | 40 | | ec:1.14.11.15 | | 3beta-dioxygenase | | -4.61 | |  | |  |
| Apaniculata8936_c0_seq1_len=385 | 85 | 9.24 | 9.68 | | ec:1.14.11.15 | | 3beta-dioxygenase (2-oxoglutarate-dependent) | | -5.56 | |  | |  |
| Apaniculata14822_c0_seq3_len=1041 | 176 | 9.03 | 19.86 | | ec:1.14.13.11 | | 4-monooxygenase | | -2.65 | |  | |  |
| Apaniculata16852_c0_seq2_len=1991 | 545 | 9.01 | 62.34 | | ec:1.14.13.11 | | 4-hydroxylase | | 0.12 | |  | |  |
| Apaniculata16893_c0_seq1_len=1928 | 536 | 9.23 | 61.37 | | ec:1.14.13.11 | | 4-monooxygenase | | 0.73 | |  | |  |
| Apaniculata26732_c0_seq1_len=681 | 162 | 9.3 | 17.76 | | ec:1.14.13.11 | | 4-monooxygenase | | -2.21 | |  | |  |
| Apaniculata30286_c0_seq1_len=2099 | 528 | 9.37 | 57.36 | | ec:1.14.13.132 | | squalene monooxygenase | | -2.39 | |  | |  |
| Apaniculata37052_c0_seq1_len=1329 | 189 | 8.64 | 20.59 | | ec:1.14.13.132 | | squalene monooxygenase | | -4.22 | | DDFSD | |  |
| Apaniculata9810_c0_seq1_len=2196 | 552 | 8.74 | 60.07 | | ec:1.14.13.132 | | squalene monooxygenase | | -2.37 | |  | |  |
| **Apaniculata13173_c0_seq1_len=1546** | **515** | **8.82** | **58.3** | | **ec:1.14.13.47** | | **3-monooxygenase** | | **-7.54** | | FGSGRRICPG; EDIVD | |  |
| Apaniculata13173_c0_seq2_len=771 | 246 | 5.73 | 27.8 | | ec:1.14.13.47 | | 3-monooxygenase | | -7.54 | |  | |  |
| Apaniculata13173_c0_seq3_len=1483 | 494 | 8.38 | 55.34 | | ec:1.14.13.47 | | 3-monooxygenase | | -6.78 | | EDIVD | |  |
| **Apaniculata19627_c0_seq2_len=843** | **268** | **5.13** | **30.85** | | **ec:1.14.13.47** | | **3-monooxygenase** | | **-6.26** | | EDIVD: EDGRD | |  |
| **Apaniculata45721_c0_seq1_len=1681** | **521** | **9.08** | **58.74** | | **ec:1.14.13.47** | | **3-monooxygenase** | | **-4.27** | | FGAGRRLCPG; EDTVD | |  |
| **Apaniculata19627_c0_seq1_len=1200** | **387** | **5.92** | **44.31** | | **ec:1.14.13.48** | | **6-monooxygenase** | | **-6.67** | | DDGRD; FGTGKRMCPG; EDIVD | |  |
| Apaniculata21906_c0_seq1_len=3380 | 383 | 6.88 | 41.95 | | ec:1.14.13.76 | | 10beta-hydroxylase | | -3.56 | |  | |  |
| **Apaniculata21906_c0_seq3_len=1606** | **457** | **9.5** | **52.51** | | **ec:1.14.13.76** | | **10beta-hydroxylase** | | **-3.58** | | EDDKD | |  |
| Apaniculata10454_c0_seq2_len=1751 | 490 | 8.77 | 55.8 | | ec:1.14.13.77 | | 13alpha-hydroxylase | | -5.79 | |  | |  |
| **Apaniculata11412_c0_seq1_len=1142** | **334** | **9.12** | **37.4** | | **ec:1.14.13.77** | | **13alpha-hydroxylase** | | **-6.07** | | FGGGTRCCPG | |  |
| Apaniculata122944_c0_seq1_len=593 | 155 | 10.02 | 18.04 | | ec:1.14.13.77 | | 13alpha-hydroxylase | | -5.88 | |  | |  |
| Apaniculata145063_c0_seq1_len=583 | 194 | 9.42 | 21.42 | | ec:1.14.13.77 | | 13alpha-hydroxylase | | -6.43 | |  | |  |
| Apaniculata16873_c0_seq4_len=302 | 87 | 9.1 | 98.29 | | ec:1.14.13.77 | | 13alpha-hydroxylase | | -6.36 | |  | |  |
| Apaniculata222742_c0_seq1_len=438 | 146 | 5.82 | 17.22 | | ec:1.14.13.77 | | 13alpha-hydroxylase (beta-amyrin 28-oxidase-like) | | -7.78 | |  | |  |
| Apaniculata566835_c0_seq1_len=258 | 86 | 5.48 | 10.05 | | ec:1.14.13.77 | | 13alpha-hydroxylase | | -8.51 | |  | |  |
| Apaniculata80271_c0_seq1_len=751 | 111 | 6.05 | 12.85 | | ec:1.14.13.77 | | 13alpha-hydroxylase | | -6.92 | |  | |  |
| Apaniculata41280_c0_seq1_len=2113 | 521 | 7.25 | 58.91 | | ec:1.14.13.78 | | ent-kaurene oxidase, chloroplastic | | -4.15 | |  | |  |
| Apaniculata23282_c0_seq1_len=1733 | 482 | 5.72 | 54.2 | | ec:1.17.1.2 | | 4-hydroxy-3-methylbut-2-enyl diphosphate reductase, chloroplastic | | 3.73 | |  | |  |
| Apaniculata24087_c1_seq1_len=1592 | 465 | 5.57 | 52.31 | | ec:1.17.1.2 | | 4-hydroxy-3-methylbut-2-enyl diphosphate reductase, chloroplastic | | 0.6 | |  | |  |
| Apaniculata18628_c1_seq1_len=2827 | 751 | 5.83 | 83.4 | | ec:1.17.7.1 | | 4-hydroxy-3-methylbut-2-enyl diphosphate synthase (ferredoxin) | | 2.61 | | DDLVID | |  |
| Apaniculata197069_c0_seq1_len=301 | 100 | 9.1 | 10.98 | | ec:1.3.3.9 | | synthase | | -7.36 | |  | |  |
| **Apaniculata23133_c0_seq17_len=2200** | **524** | **9.35** | **59.35** | | **ec:1.3.3.9** | | **synthase** | | **-3.82** | | FGLGARRCIG | |  |
| Apaniculata445953_c0_seq1_len=201 | 66 | 8.55 | 72.68 | | ec:1.3.3.9 | | synthase | | -8.57 | |  | |  |
| Apaniculata19861_c0_seq6_len=1211 | 75 | 5.02 | 8.38 | | ec:1.6.5.2 | | dehydrogenase (quinone) (thiol-disulfide oxidoreductase ) | | -4.13 | |  | |  |
| Apaniculata33088_c0_seq1_len=1774 | 502 | 6.19 | 56.26 | | ec:1.8.3.5 | | farnesylcysteine lyase | | -2.81 | |  | |  |
| Apaniculata17729_c0_seq5_len=2119 | 231 | 8.3 | 26.94 | | ec:2.1.1.100 | | S-isoprenylcysteine O-methyltransferase | | -4.23 | |  | |  |
| Apaniculata165030_c0_seq1_len=1219 | 224 | 8.33 | 26.34 | | ec:2.1.1.100 | | S-isoprenylcysteine O-methyltransferase A | | -6.39 | |  | |  |
| Apaniculata1783_c0_seq1_len=1263 | 312 | 6.82 | 34.33 | | ec:2.1.1.114 | | hexaprenyldihydroxybenzoate methyltransferase | | -3.57 | |  | |  |
| Apaniculata26878_c0_seq1_len=1495 | 377 | 7.73 | 41.08 | | ec:2.1.1.95 | | tocopherol O-methyltransferase | | -1.37 | |  | |  |
| Apaniculata16523_c0_seq4_len=3321 | 727 | 6.43 | 78.53 | | ec:2.2.1.7 | | 1-deoxy-D-xylulose-5-phosphate synthase | | 0.1 | |  | |  |
| Apaniculata239911_c0_seq1_len=442 | 147 | 7.16 | 15.8 | | ec:2.2.1.7 | | 1-deoxy-D-xylulose-5-phosphate synthase | | -7.45 | |  | |  |
| Apaniculata14374_c0_seq1_len=2603 | 740 | 6.71 | 81.34 | | ec:2.2.1.7 | | 1-deoxy-D-xylulose-5-phosphate synthase, chloroplastic | | -4.7 | |  | |  |
| Apaniculata177819_c0_seq1_len=430 | 143 | 6.93 | 15.62 | | ec:2.2.1.7 | | 1-deoxy-D-xylulose-5-phosphate synthase 2, chloroplastic | | -7.03 | |  | |  |
| Apaniculata127834_c0_seq1_len=954 | 291 | 5.99 | 31.57 | | ec:2.2.1.7 | | 1-deoxy-D-xylulose-5-phosphate synthase 2, chloroplastic | | -7.05 | |  | |  |
| Apaniculata18578_c0_seq1_len=3004 | 721 | 7.71 | 77.25 | | ec:2.2.1.7 | | 1-deoxy-D-xylulose-5-phosphate synthase 2, chloroplastic | | -3.04 | | NSE | |  |
| Apaniculata23346_c0_seq1_len=2691 | 785 | 7 | 85 | | ec:2.2.1.7 | | 1-deoxy-D-xylulose-5-phosphate synthase | | 2.66 | |  | |  |
| Apaniculata19814_c0_seq12_len=5701 | 1700 | 6.21 | 186.93 | | ec:2.2.1.9 | | synthase | | -4.72 | | DEFDD | |  |
| Apaniculata48396_c0_seq1_len=391 | 130 | 10.14 | 14.68 | | ec:2.3.1.167 | | 10-O-acetyltransferase | | -4.03 | |  | |  |
| Apaniculata10921_c0_seq4_len=1964 | 442 | 8.7 | 46.3 | | ec:2.3.1.9 | | acetyl-CoA acetyltransferase, cytosolic | | -2.2 | |  | |  |
| Apaniculata8743_c0_seq1_len=1899 | 408 | 6.36 | 41.93 | | ec:2.3.1.9 | | acetyl-CoA acetyltransferase, cytosolic | | -1.78 | |  | |  |
| Apaniculata15235_c0_seq1_len=1908 | 473 | 5.98 | 52.33 | | ec:2.3.3.10 | | hydroxymethylglutaryl-CoA synthase | | -1.11 | |  | |  |
| Apaniculata9938_c0_seq4_len=1528 | 156 | 5.11 | 17.91 | | ec:2.5.1.1 | | geranyl-diphosphate synthase | | -4.93 | | DDYLD | |  |
| Apaniculata20395_c0_seq1_len=3925 | 315 | 7.67 | 35.68 | | ec:2.5.1.1 | | geranyl-diphosphate synthase | | -2.62 | | NSE | |  |
| Apaniculata455521_c0_seq1_len=264 | 86 | 7.96 | 10.01 | | ec:2.5.1.10 | | 3-ketoacyl-ACP diphosphate synthase | | -8.78 | |  | |  |
| Apaniculata4885_c0_seq1_len=1661 | 375 | 5.69 | 43.19 | | ec:2.5.1.10 | | farnesyl pyrophosphate synthase | | -0.86 | | DDIMD; DDYLD | |  |
| Apaniculata24554_c0_seq1_len=1469 | 353 | 5.18 | 37.89 | | ec:2.5.1.10 | | geranylgeranyl pyrophosphate synthase, chloroplastic | | -0.31 | | DNDD; DDILD | |  |
| Apaniculata6102_c0_seq1_len=1458 | 405 | 5.74 | 43.5 | | ec:2.5.1.10 | | geranylgeranyl pyrophosphate synthase, chloroplastic | | 1.76 | | DNDD; DDILD | |  |
| Apaniculata7735_c0_seq1_len=750 | 224 | 8.24 | 24.81 | | ec:2.5.1.10 | | diphosphate synthase | | -3.47 | |  | |  |
| Apaniculata473514_c0_seq1_len=243 | 81 | 4.64 | 84.47 | | ec:2.5.1.10 | | GGPP diphosphate synthase | | -8.71 | |  | |  |
| Apaniculata177727_c0_seq1_len=707 | 152 | 8.96 | 17.52 | | ec:2.5.1.21 | | squalene synthase | | -7.75 | | EDFKD | |  |
| Apaniculata180044_c0_seq1_len=624 | 207 | 5.75 | 24.49 | | ec:2.5.1.21 | | squalene synthase | | -7.82 | | DDYDE | |  |
| Apaniculata7704_c1_seq1_len=1601 | 421 | 6.62 | 48.12 | | ec:2.5.1.21 | | squalene synthase | | -0.8 | | DDYDE | |  |
| Apaniculata7707_c0_seq1_len=2349 | 383 | 7.28 | 41.57 | | ec:2.5.1.29 | | diphosphate synthase | | -1.97 | | DDILD | |  |
| Apaniculata46074_c0_seq1_len=658 | 180 | 9.27 | 19.34 | | ec:2.5.1.29 | | GGPS diphosphate synthase | | -4.04 | |  | |  |
| Apaniculata7952_c0_seq1_len=1610 | 438 | 6.3 | 50.71 | | ec:2.5.1.29 | | squalene diphosphate synthase | | -6.51 | |  | |  |
| Apaniculata1252_c0_seq1_len=614 | 204 | 6.08 | 23.46 | | ec:2.5.1.29 | | squalene diphosphate synthase | | -6.33 | | DDYDE | |  |
| Apaniculata1252_c0_seq2_len=1570 | 429 | 7.24 | 48.87 | | ec:2.5.1.29 | | squalene diphosphate synthase | | -7.22 | | EDVRD; DTE | |  |
| Apaniculata6946_c0_seq1_len=1798 | 255 | 7.03 | 29.01 | | ec:2.5.1.30 | | solanesyl-diphosphate synthase 3, chloroplastic | | -3.98 | | DDVLD | |  |
| Apaniculata6895_c0_seq1_len=1745 | 424 | 6.38 | 47.49 | | ec:2.5.1.30 | | solanesyl-diphosphate synthase 3, chloroplastic | | -3.76 | | DDVLD; DDILD | |  |
| Apaniculata13979_c0_seq1_len=3595 | 436 | 6.24 | 48.09 | | ec:2.5.1.30 | | diphosphate synthase | | -0.01 | | EDDDE | |  |
| Apaniculata15668_c0_seq4_len=1941 | 441 | 6.93 | 49.49 | | ec:2.5.1.32 | | phytoene synthase 2, chloroplastic | | -1.55 | |  | |  |
| Apaniculata32857_c0_seq1_len=1178 | 280 | 6.93 | 31.24 | | ec:2.5.1.32 | | phytoene synthase 2, chloroplastic | | -2.82 | |  | |  |
| Apaniculata22922_c0_seq34_len=1406 | 355 | 4.93 | 41.47 | | ec:2.5.1.58 | | farnesyltransferase/geranylgeranyltransferase | | -3.21 | |  | |  |
| Apaniculata20503_c0_seq3_len=890 | 142 | 5.07 | 15.97 | | ec:2.5.1.84 | | solanesyl diphosphate synthase 3, chloroplastic/mitochondrial | | -3.22 | | DDVLD | |  |
| Apaniculata15292_c0_seq3_len=1344 | 398 | 6.24 | 44.35 | | ec:2.6.1.5 | | aspartate-prephenate aminotransferase | | -2.06 | |  | |  |
| Apaniculata17769_c0_seq1_len=1389 | 96 | 9.1 | 11.08 | | ec:2.6.1.5 | | transaminase | | -6.77 | |  | |  |
| Apaniculata17769_c0_seq2_len=2558 | 448 | 9.1 | 49.16 | | ec:2.6.1.5 | | Aspartate aminotransferase 3 | | -4.98 | |  | |  |
| Apaniculata23002_c0_seq13_len=1686 | 443 | 6.01 | 49.08 | | ec:2.6.1.5 | | histidinol-phosphate aminotransferase, chloroplastic | | -3.6 | |  | |  |
| Apaniculata240443_c0_seq1_len=705 | 138 | 4.79 | 15.22 | | ec:2.6.1.5 | | transaminase | | -8.45 | | NSE | |  |
| Apaniculata272438_c0_seq1_len=594 | 197 | 8.41 | 22 | | ec:2.6.1.5 | | kynurenine--oxoglutarate transaminase 1 | | -7.94 | |  | |  |
| Apaniculata27983_c0_seq1_len=1869 | 478 | 8.14 | 52.44 | | ec:2.6.1.5 | | kynurenine--oxoglutarate transaminase 1 | | -1.76 | |  | |  |
| Apaniculata33488_c0_seq1_len=2053 | 485 | 7.76 | 51.98 | | ec:2.6.1.5 | | bifunctional aspartate aminotransferase | | -3.22 | |  | |  |
| Apaniculata4151_c0_seq1_len=1690 | 349 | 8.41 | 39.27 | | ec:2.6.1.5 | | transaminase | | -1.98 | |  | |  |
| Apaniculata4978_c0_seq1_len=2344 | 408 | 7.82 | 44.13 | | ec:2.6.1.5 | | transaminase | | -2.55 | |  | |  |
| Apaniculata52435_c0_seq1_len=1674 | 415 | 6.6 | 45.91 | | ec:2.6.1.5 | | kynurenine--oxoglutarate transaminase 1 | | -4.19 | |  | |  |
| Apaniculata7700_c0_seq1_len=2046 | 489 | 8.14 | 54.07 | | ec:2.6.1.5 | | transaminase | | -1.36 | |  | |  |
| Apaniculata9075_c0_seq2_len=2027 | 331 | 7.04 | 36.66 | | ec:2.6.1.5 | | transaminase | | -1.33 | | DDID | |  |
| Apaniculata9090_c0_seq2_len=1622 | 244 | 7.14 | 26.72 | | ec:2.6.1.5 | | transaminase | | -5.28 | |  | |  |
| Apaniculata220_c0_seq1_len=1144 | 377 | 8.37 | 41.82 | | ec:2.6.1.83 | | aminotransferase | | -7.25 | | DTE | |  |
| Apaniculata220_c0_seq2_len=3093 | 444 | 8.11 | 49.08 | | ec:2.6.1.83 | | aminotransferase | | -6.69 | | DTE | |  |
| Apaniculata25701_c0_seq1_len=1853 | 485 | 7.17 | 52.86 | | ec:2.6.1.83 | | aminotransferase | | -0.89 | |  | |  |
| Apaniculata26494_c0_seq1_len=1581 | 430 | 6.2 | 47.55 | | ec:2.7.1.148 | | 4-diphosphocytidyl-2-C-methyl-D-erythritol kinase, chloroplastic | | -1.04 | |  | |  |
| Apaniculata5343_c0_seq2_len=1637 | 396 | 6.04 | 41.93 | | ec:2.7.1.36 | | mevalonate kinase | | -3.14 | |  | |  |
| Apaniculata2931_c0_seq1_len=1922 | 509 | 5.03 | 55.02 | | ec:2.7.4.2 | | phosphomevalonate kinase | | -4.02 | |  | |  |
| Apaniculata22179_c0_seq19_len=2679 | 593 | 6.39 | 64.16 | | ec:2.7.4.2 | | phosphomevalonate kinase | | -6.32 | |  | |  |
| Apaniculata24573_c0_seq1_len=1282 | 341 | 7.22 | 37.61 | | ec:2.7.7.60 | | 2-C-methyl-D-erythritol 4-phosphate cytidylyltransferase, chloroplastic | | 0.07 | | NSE | |  |
| Apaniculata65951_c0_seq1_len=1431 | 476 | 7.12 | 54.86 | | ec:3.1.7.3 | | bornyl pyrophosphate hydrolase (geraniol synthase, chloroplastic-like) | | -4.66 | | DDVFD | |  |
| Apaniculata27915_c0_seq1_len=1628 | 436 | 6.56 | 48.1 | | ec:4.1.1.33 | | mevalonate diphosphate decarboxylase | | -1.73 | |  | |  |
| Apaniculata4462_c0_seq2_len=1312 | 382 | 9.01 | 41.87 | | ec:4.1.3.36 | | 1,4-dihydroxy-2-naphthoyl-CoA synthase, peroxisomal | | -1.34 | | DARDD | |  |
| Apaniculata12753_c0_seq1_len=1917 | 570 | 6.92 | 65.97 | | ec:4.2.3.15 | | tricyclene synthase 0e23, chloroplastic | | -3.46 | | DDVFD | |  |
| Apaniculata595796_c0_seq1_len=227 | 75 | 5.19 | 8.87 | | ec:4.2.3.26 | | geraniol synthase, chloroplastic | | -8.94 | |  | |  |
| Apaniculata76098_c0_seq1_len=443 | 107 | 5.51 | 12.44 | | ec:4.2.3.26 | | geraniol synthase, chloroplastic | | -4.97 | |  | |  |
| Apaniculata82683_c0_seq1_len=745 | 247 | 5.17 | 28.74 | | ec:4.2.3.26 | | geraniol synthase, chloroplastic | | -5.77 | | DDVFD | |  |
| Apaniculata23661_c0_seq1_len=1049 | 241 | 8.83 | 25.39 | | ec:4.6.1.12 | | 2-C-methyl-D-erythritol 2,4-cyclodiphosphate synthase, chloroplastic | | 1.38 | |  | |  |
| Apaniculata81028_c0_seq1_len=528 | 176 | 9.1 | 19.2 | | ec:5.3.3.2 | | isopentenyl diphosphate delta-isomerase | | -5.31 | |  | |  |
| Apaniculata24192_c0_seq1_len=1239 | 309 | 6.67 | 35.42 | | ec:5.3.3.2 | | isopentenyl diphosphate delta-isomerase | | -0.81 | |  | |  |
| Apaniculata18258_c0_seq2_len=3578 | 295 | 6.48 | 32.61 | | ec:5.4.4.2 | | isochorismate synthase | | -4.49 | |  | |  |
| Apaniculata18258_c0_seq3_len=3974 | 323 | 6.09 | 35.57 | | ec:5.4.4.2 | | isochorismate synthase | | -4.6 | |  | |  |
| Apaniculata62744_c0_seq1_len=1561 | 452 | 8.45 | 50.54 | | ec:5.4.4.2 | | Isochorismate synthase, chloroplastic | | -4.92 | | DTE | |  |
| Apaniculata313812_c0_seq1_len=266 | 88 | 5.68 | 96.13 | | ec:5.5.1.12 | | copalyl diphosphate synthase 3 | | -7.86 | |  | |  |
| Apaniculata135474_c0_seq1_len=1721 | 573 | 8.52 | 66.52 | | ec:5.5.1.13 | | ent-copaly diphosphate synthase | | -7.1 | | DVDD | |  |
| Apaniculata13895_c0_seq2_len=2201 | 533 | 6.6 | 57.36 | | ec:6.2.1.12 | | oxalate--CoA ligase | | 2.46 | |  | |  |
| Apaniculata15744_c0_seq1_len=2969 | 581 | 6.01 | 63.47 | | ec:6.2.1.12 | | ligase | | -0.68 | | DGDD | |  |
| Apaniculata16438_c0_seq2_len=759 | 121 | 9.87 | 12.91 | | ec:6.2.1.12 | | ligase | | -0.1 | |  | |  |
| Apaniculata22108_c0_seq2_len=705 | 130 | 5.07 | 14.62 | | ec:6.2.1.12 | | ligase 2 | | -5.72 | |  | |  |
| Apaniculata22108_c0_seq6_len=1099 | 115 | 8.7 | 12.92 | | ec:6.2.1.12 | | ligase 2 | | -4.26 | |  | |  |
| Apaniculata2328_c0_seq2_len=1847 | 570 | 5.18 | 61.87 | | ec:6.2.1.12 | | ligase | | -3.36 | | DDEFD; DTE | |  |
| Apaniculata28775_c0_seq1_len=877 | 239 | 6.27 | 26.04 | | ec:6.2.1.12 | | ligase | | -1.76 | | DTE | |  |
| Apaniculata31782_c0_seq1_len=1051 | 326 | 5.44 | 35.55 | | ec:6.2.1.12 | | ligase | | -2.41 | |  | |  |
| Apaniculata6793_c0_seq2_len=5272 | 644 | 8.77 | 71.88 | | ec:6.2.1.12 | | ligase | | -0.95 | |  | |  |
| Apaniculata14367_c0_seq1_len=2448 | 364 | 7.41 | 40.24 | | ec:6.2.1.26 | | malonate--CoA ligase | | -3.46 | |  | |  |
| Apaniculata26433_c0_seq1_len=2138 | 565 | 7.97 | 62.28 | | ec:6.2.1.26 | | ligase (peroxisomal) | | -1.19 | |  | |  |
| Apaniculata7770_c0_seq1_len=1944 | 572 | 8.37 | 62.24 | | ec:6.2.1.26 | | ligase (acyl-activating enzyme 5, peroxisomal ) | | -0.92 | |  | |  |
| Apaniculata81917_c0_seq1_len=436 | 91 | 12.16 | | 10.9 | | ec:6.2.1.26 | | ligase | | -5.79 | |  | |
| Apaniculata85453_c0_seq1_len=1549 | 495 | 6.49 | | 53.61 | | ec:6.2.1.26 | | ligase (acyl-activating enzyme 5, peroxisomal ) | | -5.19 | | NSE | |
