# Supplementary material for: De novo Assembly of Leaf Transcriptome in the Medicinal Plant Andrographis paniculata
Source: Front Plant Sci. 2016 Aug 17;7:1203. doi: 10.3389/fpls.2016.01203 (PMC4987368; doi:10.3389/fpls.2016.01203)
Supplement: Supplementary File S7 — Family wise distribution of cytochrome P450 transcripts expressed in the leaf of A. paniculata. [file Table7.docx]

| **Supplementary File S7. Family wise distribution of cytochrome P450 transcripts expressed in the leaf of *A.paniculata*** | |
| --- | --- |
| **CYP clan and family name** | **No. of genes** |
| **CYP71 CLAN** |  |
| CYP703 | 1 |
| CYP706 | 2 |
| CYP71 | 17 |
| CYP736 | 6 |
| CYP76 | 11 |
| CYP77 | 1 |
| CYP78 | 5 |
| CYP79 | 1 |
| CYP81 | 9 |
| CYP82 | 8 |
| CYP84 | 1 |
| CYP92 | 1 |
| CYP98 | 1 |
| **CYP72 CLAN** |  |
| CYP72 | 13 |
| CYP714 | 3 |
| CYP721 | 1 |
| CYP734 | 1 |
| CYP749 | 2 |
| **CYP85 CLAN** |  |
| CYP707 | 5 |
| CYP716 | 3 |
| CYP720 | 1 |
| CYP722 | 1 |
| CYP724 | 1 |
| CYP733 | 1 |
| CYP85 | 2 |
| CYP87 | 2 |
| CYP90 | 3 |
| **CYP86 CLAN** |  |
| CYP86 | 4 |
| CYP704 | 4 |
| CYP94 | 5 |
| CYP96 | 4 |
| **CYP711 CLAN** |  |
| CYP711 | 1 |
| **CYP727 CLAN** |  |
| CYP727 | 1 |
| **CYP97 CLAN** |  |
| CYP97 | 2 |
| **TOTAL** | **124** |
