# Supplementary material for: De novo Assembly of Leaf Transcriptome in the Medicinal Plant Andrographis paniculata
Source: Front Plant Sci. 2016 Aug 17;7:1203. doi: 10.3389/fpls.2016.01203 (PMC4987368; doi:10.3389/fpls.2016.01203)
Supplement: Supplementary File S8 — Classification and properties of deduced Cytochrome P450s in A. paniculata leaf transcriptome. [file Table8.docx]

| **Supplementary File S8. Classification and properties of deduced Cytochrome P450s in *A.paniculata* leaf transcriptome** | | | | | | | | | |
| --- | --- | --- | --- | --- | --- | --- | --- | --- | --- |
| **Transcript_ID** | **CYP Name** | **Protein Length** | **PI** | **MW** | **Read Count** | **RPKM** | **Fold Expression** | **Relative Expression** | **CYP Motif Sequence** |
| Apaniculata52043_c0_seq1_len=1690 | >CYP703A24 | 516 | 6.69 | 58710.51 | 1131 | 3.54 | 1.83 | -4.56 | FSAGKRKCPG |
| Apaniculata20957_c0_seq22_len=1575 | >CYP704A102 | 504 | 9.02 | 57226.12 | 99 | 0.33218 | -1.589974 | -8.31 |  |
| Apaniculata21588_c0_seq31_len=1246 | >CYP704A102as | 374 | 8.52 | 42413.94 | 120 | 0.51 | -0.98 | -7.36 |  |
| Apaniculata15905_c0_seq1_len=1862 | >CYP704A103 | 367 | 6.05 | 41726.27 | 1219 | 3.45971 | 1.790651 | -4.93 |  |
| Apaniculata376720_c0_seq1_len=574 | >CYP704B-fragment1 | 75 | 9.53 | 11543.4 | 30 | 0.28 | -1.84 | -8.22 |  |
| Apaniculata22702_c1_seq3_len=1331 | >CYP706G-fragment1 | 189 | 5.54 | 23300.98 | 49820 | 197.81 | 7.63 | 1.25 | FGSGRRICVG |
| Apaniculata23911_c0_seq1_len=771 | >CYP706G-fragment3 | 232 | 9.48 | 28658.31 | 10045 | 68.86 | 6.11 | -0.28 |  |
| Apaniculata21523_c0_seq8_len=1741 | >CYP707A108 | 408 | 8.77 | 46957.66 | 303 | 0.91973 | -0.120721 | -6.84 |  |
| Apaniculata102653_c0_seq1_len=1606 | >CYP707A109 | 486 | 9.02 | 54493.53 | 371 | 1.2208 | 0.2878251 | -6.43 | FGSGVHACPG |
| Apaniculata25380_c0_seq1_len=1731 | >CYP707A110 | 487 | 9.3 | 55326.12 | 20120 | 61.4252 | 5.9407586 | -0.78 | FGNGVHSCPG |
| Apaniculata13701_c0_seq2_len=1128 | >CYP707A111 | 475 | 9.1 | 55120.1 | 2102 | 9.8478 | 3.2998015 | -3.42 | FGSGAHSCPG |
| Apaniculata21594_c0_seq22_len=1546 | >CYP707A112 | 284 | 8.7 | 32889 | 558 | 1.90739 | 0.9316026 | -5.79 | FGSGIHSCPG |
| Apaniculata20531_c0_seq2_len=1929 | >CYP711A51 | 460 | 8.61 | 51282.99 | 1707 | 4.68 | 2.23 | -4.16 |  |
| Apaniculata13542_c0_seq1_len=763 | >CYP714A26 | 155 | 9.6 | 17527.75 | 305 | 2.11247 | 1.0789321 | -5.64 | FGLGPRLCLG |
| Apaniculata2623_c0_seq2_len=1640 | >CYP714A28 | 510 | 9.44 | 58062.71 | 2322 | 7.49 | 2.91 | -3.48 | FGLGPRLCLG |
| Apaniculata166285_c0_seq1_len=958 | >CYP714G16 | 280 | 8.57 | 33310.66 | 104 | 0.58 | -0.79 | -7.17 | FSFGPRVCVG |
| Apaniculata85888_c0_seq1_len=1524 | >CYP716A90 | 476 | 8.72 | 54398.21 | 450 | 1.56043 | 0.6419399 | -6.08 | FGGGPRMCPG |
| Apaniculata195793_c0_seq1_len=1211 | >CYP716A95 | 365 | 9.15 | 45348.56 | 84 | 0.37 | -1.44 | -7.82 | FGGGPRMCPG |
| Apaniculata17508_c0_seq3_len=1900 | >CYP716D26 | 334 | 10.01 | 37653.41 | 927 | 2.57835 | 1.3664477 | -5.35 |  |
| Apaniculata10611_c0_seq3_len=2064 | >CYP71A62 | 509 | 6.59 | 60169.46 | 5074 | 13 | 3.71 | -2.69 | FGAGRRGCPG |
| Apaniculata111988_c0_seq1_len=1603 | >CYP71AH17 | 499 | 8.72 | 56741.51 | 493 | 1.62528 | 0.700691 | -6.02 | FGAGRRQCPG |
| Apaniculata5604_c0_seq2_len=1769 | >CYP71AP30 | 513 | 6.76 | 59942.56 | 3594 | 10.74 | 3.43 | -2.96 | FGAGRRSCPA |
| Apaniculata249704_c0_seq1_len=328 | >CYP71AT95 | 109 | 6.33 | 12679.74 | 29 | 0.46724 | -1.097765 | -7.81 |  |
| Apaniculata22741_c0_seq3_len=1757 | >CYP71AU63 | 545 | 6.52 | 61397.08 | 469 | 1.41064 | 0.4963516 | -6.22 | FGAGRRSCPG |
| Apaniculata22900_c0_seq2_len=1657 | >CYP71AU64 | 352 | 7.01 | 39857.15 | 1109 | 3.53692 | 1.8224917 | -4.89 |  |
| Apaniculata23339_c0_seq1_len=3332 | >CYP71BE50 | 499 | 8.13 | 55626.43 | 323293 | 512.751 | 9.002116 | 2.3 | FGAGRRICPG |
| Apaniculata29675_c0_seq1_len=413 | >CYP71BS-fragment1 | 134 | 9.98 | 15376.82 | 1387 | 17.75 | 4.15 | -2.24 |  |
| Apaniculata71977_c0_seq1_len=1373 | >CYP71CF1 | 372 | 5.44 | 41276.91 | 473 | 1.82057 | 0.8643864 | -5.85 | FGGGRRGCPG |
| Apaniculata7036_c0_seq1_len=1653 | >CYP71CG1 | 501 | 8.67 | 56472.47 | 493 | 1.57612 | 0.6563787 | -6.06 | FGAGRRGCPG |
| Apaniculata127964_c0_seq1_len=988 | >CYP71CH1 | 305 | 9.3 | 34568.53 | 119 | 0.63651 | -0.651744 | -7.37 |  |
| Apaniculata13173_c0_seq1_len=1546 | >CYP71D439 | 509 | 8.71 | 57709.85 | 129 | 0.44096 | -1.181291 | -7.9 | FGSGRRICPG |
| Apaniculata154763_c0_seq1_len=329 | >CYP71D440 | 98 | 8.88 | 11406.11 | 33 | 0.53007 | -0.915744 | -7.63 |  |
| Apaniculata21104_c0_seq1_len=1821 | >CYP71D451 | 506 | 8.63 | 59849.07 | 5058 | 14.68 | 3.88 | -2.51 | FGGGRRICPG |
| Apaniculata15682_c0_seq3_len=1293 | >CYP71D452 | 356 | 6.26 | 43078.64 | 7729 | 31.59 | 4.99 | -1.41 | FGAGRRICPG |
| Apaniculata19627_c0_seq1_len=1200 | >CYP71D453 | 377 | 5.92 | 44312.13 | 184 | 0.82 | -0.29 | -6.67 | FGTGKRMCPG |
| Apaniculata45721_c0_seq1_len=1681 | >CYP71D454 | 506 | 9.08 | 58737.64 | 1377 | 4.33 | 2.12 | -4.27 | FGAGRRLCPG |
| Apaniculata21570_c0_seq20_len=1031 | >CYP720A1 | 474 | 8.86 | 54130.79 | 62 | 0.3178 | -1.653826 | -8.37 | FGGGGRFCPG |
| Apaniculata14227_c0_seq2_len=1795 | >CYP721A48 | 508 | 9.3 | 60865.5 | 1028 | 3.03 | 1.6 | -4.79 | FGLGHRVCVG |
| Apaniculata17179_c0_seq1_len=2185 | >CYP722C6 | 430 | 9.09 | 49272.08 | 1533 | 3.70771 | 1.8905303 | -4.83 |  |
| Apaniculata11412_c0_seq1_len=1142 | >CYP724B25 | 489 | 9.22 | 55508.38 | 269 | 1.24481 | 0.3159213 | -6.4 | FGGGTRCCPG |
| Apaniculata16822_c0_seq1_len=2040 | >CYP727B12 | 372 | 8.23 | 41360.03 | 990 | 2.56461 | 1.3587372 | -5.36 |  |
| Apaniculata70172_c0_seq1_len=1605 | >CYP72A342 | 461 | 8.38 | 52968.7 | 673 | 2.21593 | 1.147911 | -5.57 | FGWGPRICIG |
| Apaniculata6223_c0_seq1_len=1889 | >CYP72A343 | 384 | 8.65 | 43922.91 | 7134 | 19.958 | 4.3188944 | -2.4 |  |
| Apaniculata108592_c0_seq1_len=1100 | >CYP72A344 | 322 | 7.78 | 36212.8 | 174 | 0.83593 | -0.258538 | -6.98 | FGAGPRLCIG |
| Apaniculata6814_c0_seq2_len=750 | >CYP72A345 | 259 | 5.8 | 29693.7 | 1133 | 7.98333 | 2.9969913 | -3.72 | FGWGPRICIG |
| Apaniculata14163_c0_seq3_len=675 | >CYP72A346 | 218 | 9.35 | 25099.2 | 564 | 4.41561 | 2.1426136 | -4.57 |  |
| Apaniculata369603_c0_seq1_len=347 | >CYP72A347 | 87 | 6.39 | 9702.33 | 34 | 0.5178 | -0.949523 | -7.67 |  |
| Apaniculata7352_c0_seq2_len=1755 | >CYP72A398 | 518 | 8.11 | 60649.48 | 923 | 2.78 | 1.48 | -4.91 |  |
| Apaniculata22614_c0_seq4_len=2015 | >CYP72A399 | 526 | 9.25 | 62579.57 | 10455 | 27.42 | 4.78 | -1.61 | FGWGPRICIG |
| Apaniculata14150_c0_seq2_len=1685 | >CYP72D15 | 515 | 9.18 | 59617.68 | 4132 | 12.96 | 3.7 | -2.69 |  |
| Apaniculata15694_c0_seq1_len=1806 | >CYP72E1 | 517 | 8.85 | 59492.65 | 9763 | 28.5681 | 4.8363326 | -1.88 | FGWGPRICLG |
| Apaniculata4733_c0_seq1_len=2023 | >CYP72F1 | 392 | 8.51 | 44773.09 | 447842 | 1169.89 | 10.192156 | 3.49 |  |
| Apaniculata18279_c0_seq3_len=1989 | >CYP72F3 | 522 | 9.28 | 62311.03 | 6789 | 18.04 | 4.18 | -2.21 | FGYGPRICVG |
| Apaniculata29512_c0_seq1_len=1867 | >CYP72G1 | 521 | 9 | 59829.57 | 5482 | 15.52 | 3.96 | -2.43 | FGWGPRICIG |
| Apaniculata352467_c0_seq1_len=297 | >CYP733A1 | 228 | 5.57 | 26288.87 | 18 | 0.32028 | -1.642588 | -8.36 |  |
| Apaniculata23133_c0_seq17_len=2200 | >CYP734A43 | 522 | 9.35 | 59350.55 | 2469 | 5.94 | 2.58 | -3.82 | FGLGARRCIG |
| Apaniculata135527_c0_seq1_len=1725 | >CYP736A143 | 503 | 8.04 | 56437.66 | 226 | 0.69236 | -0.530396 | -7.25 | FGSGRRSCPG |
| Apaniculata159650_c0_seq1_len=1339 | >CYP736A144 | 401 | 8.3 | 44821.79 | 115 | 0.45387 | -1.139644 | -7.86 | FGFGRRSCPG |
| Apaniculata7275_c0_seq2_len=1438 | >CYP736A145 | 222 | 5.37 | 25035.98 | 243 | 0.89302 | -0.16323 | -6.88 | FGSGRRGCPG |
| Apaniculata95350_c0_seq1_len=876 | >CYP736A146 | 188 | 9.63 | 21179.87 | 156 | 0.9411 | -0.087579 | -6.8 |  |
| Apaniculata28350_c0_seq1_len=1754 | >CYP736A165 | 493 | 7.07 | 55542.42 | 7375 | 22.23 | 4.48 | -1.91 | FGSGRRGCPG |
| Apaniculata58729_c0_seq1_len=1762 | >CYP736A166 | 489 | 8.79 | 55617.76 | 1137 | 3.42 | 1.78 | -4.61 | FGSGRRICPG |
| Apaniculata15212_c0_seq6_len=3659 | >CYP749A43 | 507 | 9.36 | 57483.84 | 10228 | 14.7722 | 3.8848087 | -2.83 | FGFGPRMCVG |
| Apaniculata218763_c0_seq1_len=1570 | >CYP749A44 | 453 | 8.64 | 51589.17 | 124 | 0.41739 | -1.260547 | -7.98 |  |
| Apaniculata11201_c0_seq2_len=1066 | >CYP76A40 | 318 | 9.23 | 36367.38 | 1958 | 9.70669 | 3.2789792 | -3.44 | QFGGHWRVL |
| Apaniculata11201_c0_seq1_len=1848 | >CYP76A41 | 516 | 9.31 | 61094.86 | 5574 | 15.94 | 4 | -2.39 | FGSGRRICVG |
| Apaniculata261764_c0_seq1_len=1128 | >CYP76AH18 | 404 | 9.45 | 45454.96 | 95 | 0.44507 | -1.16789 | -7.88 |  |
| Apaniculata78141_c0_seq1_len=1590 | >CYP76AL1 | 427 | 6.1 | 48337.06 | 575 | 1.91111 | 0.934413 | -5.78 | FGSGRRICPG |
| Apaniculata13878_c0_seq1_len=949 | >CYP76AM1 | 271 | 9.38 | 30040.68 | 2662 | 14.8237 | 3.8898365 | -2.83 |  |
| Apaniculata20448_c0_seq1_len=1624 | >CYP76B62 | 491 | 8.99 | 55128.1 | 2497 | 8.12546 | 3.0224501 | -3.69 | FGAGRRICPG |
| Apaniculata21261_c0_seq4_len=1656 | >CYP76B63 | 481 | 9.02 | 53076.44 | 960 | 3.06356 | 1.6152096 | -5.1 | FGGGRRICPG |
| Apaniculata387746_c0_seq1_len=420 | >CYP76G-fragment1 | 120 | 8.86 | 14860.32 | 18 | 0.23 | -2.13 | -8.51 | FGAGRRICPA |
| Apaniculata368733_c0_seq1_len=262 | >CYP76G-fragment2 | 66 | 8.09 | 10330.67 | 21 | 0.43 | -1.22 | -7.61 |  |
| Apaniculata9262_c0_seq1_len=793 | >CYP76S10 | 257 | 5.11 | 29072.23 | 795 | 5.29797 | 2.4054399 | -4.31 |  |
| Apaniculata7664_c0_seq1_len=1779 | >CYP76S9 | 494 | 7.18 | 55308.14 | 8767 | 26.043 | 4.7028227 | -2.01 | FGAGRRICPG |
| Apaniculata19040_c0_seq1_len=2107 | >CYP77A32 | 512 | 9.29 | 59665.26 | 3196 | 8.02 | 3.01 | -3.38 | FGVGRRICPG |
| Apaniculata103675_c0_seq1_len=1664 | >CYP78A127 | 524 | 9.27 | 58757.44 | 302 | 0.95911 | -0.060229 | -6.78 | FGLGRRSCPG |
| Apaniculata21698_c0_seq1_len=2123 | >CYP78A128 | 538 | 8.96 | 60227.5 | 2514 | 6.25793 | 2.6456862 | -4.07 | FGSGRRSCPG |
| Apaniculata17219_c0_seq1_len=1959 | >CYP78A171 | 531 | 9.54 | 64719.17 | 4651 | 12.55 | 3.65 | -2.74 | FGSGRRVCPG |
| Apaniculata63979_c0_seq1_len=1870 | >CYP78A172 | 547 | 8.82 | 61417.22 | 972 | 2.75 | 1.46 | -4.93 | FGAGRRVCPG |
| Apaniculata21715_c1_seq2_len=1114 | >CYP79D47 | 335 | 9.31 | 38633.27 | 508 | 2.40987 | 1.2689571 | -5.45 |  |
| Apaniculata5495_c0_seq1_len=1062 | >CYP81B69 | 501 | 8.89 | 55819 | 1054 | 5.24483 | 2.390897 | -4.33 | FGSGRRGCPG |
| Apaniculata12244_c0_seq3_len=2174 | >CYP81B70 | 332 | 9.63 | 39201.82 | 735 | 1.78667 | 0.8372701 | -5.88 |  |
| Apaniculata182734_c0_seq1_len=334 | >CYP81D-fragment1 | 104 | 11.6 | 11816.92 | 48 | 0.75947 | -0.396936 | -7.11 |  |
| Apaniculata21860_c1_seq2_len=1224 | >CYP81Q47 | 320 | 8.71 | 35225.75 | 269 | 1.16141 | 0.2158804 | -6.5 | FGMGRRACPG |
| Apaniculata21137_c1_seq2_len=3318 | >CYP81Q62 | 346 | 8.04 | 56768.51 | 2637 | 4.21 | 2.08 | -4.31 | FGMGRRACPG |
| Apaniculata21245_c0_seq2_len=1738 | >CYP81Q63 | 508 | 9.26 | 58543.82 | 5263 | 16.01 | 4.01 | -2.39 | FGMGRRSCPG |
| Apaniculata383597_c0_seq1_len=763 | >CYP81Q64a | 242 | 9.63 | 28829.63 | 42 | 0.3 | -1.74 | -8.13 |  |
| Apaniculata222165_c0_seq1_len=613 | >CYP81Q64b | 200 | 8.35 | 23096.83 | 55 | 0.48 | -1.06 | -7.45 | FGMGRRACPG |
| Apaniculata117018_c0_seq1_len=1705 | >CYP81Q65 | 499 | 5.81 | 56861.49 | 205 | 0.64 | -0.65 | -7.03 | FGSGRRKCPG |
| Apaniculata22558_c0_seq1_len=1788 | >CYP82AH1 | 513 | 8.03 | 57874.07 | 3129 | 9.25 | 3.21 | -3.18 | FGGGRRICPG |
| Apaniculata22439_c0_seq1_len=1018 | >CYP82AH2 | 287 | 5.32 | 34973.32 | 1287 | 6.69 | 2.75 | -3.65 | FGGGRRICPG |
| Apaniculata40806_c0_seq1_len=1835 | >CYP82AJ1 | 510 | 6.67 | 61533.17 | 1900 | 5.48 | 2.46 | -3.93 | FGGGRRVCPG |
| Apaniculata107473_c0_seq1_len=1724 | >CYP82AK1 | 535 | 8.05 | 61672.29 | 427 | 1.31 | 0.39 | -6 | FGGGRRICPA |
| Apaniculata145224_c0_seq1_len=1669 | >CYP82D114 | 473 | 7.79 | 58000.2 | 371 | 1.18 | 0.24 | -6.15 | FGGGRRICPG |
| Apaniculata6958_c0_seq1_len=2123 | >CYP82D115 | 528 | 9.3 | 59487.83 | 1507 | 3.76 | 1.92 | -4.48 | FGAGRRSCPG |
| Apaniculata23704_c0_seq1_len=1887 | >CYP82D116 | 519 | 8.96 | 59960.41 | 68152 | 190.87 | 7.58 | 1.2 | FGMGRRVCPG |
| Apaniculata19099_c0_seq2_len=2163 | >CYP82V3 | 338 | 9.23 | 37795.02 | 1731 | 4.22918 | 2.080378 | -4.64 |  |
| Apaniculata42365_c0_seq1_len=1750 | >CYP84A71 | 504 | 6.21 | 58099.5 | 1446 | 4.37 | 2.13 | -4.26 | FGSGRRSCPG |
| Apaniculata22789_c0_seq94_len=1193 | >CYP85A1v1 | 383 | 8.24 | 44186.86 | 135 | 0.59801 | -0.741757 | -7.46 | FGGGTRQCPG |
| Apaniculata22752_c0_seq2_len=1810 | >CYP85A1v2 | 382 | 8.7 | 43934.63 | 207 | 0.60438 | -0.726481 | -7.44 | FGGGTRQCPG |
| Apaniculata110996_c0_seq1_len=1115 | >CYP86A114 | 318 | 9.11 | 37797.93 | 241 | 1.15 | 0.21 | -6.19 |  |
| Apaniculata22328_c0_seq1_len=2626 | >CYP86A95 | 539 | 7.97 | 61253.97 | 12765 | 25.6887 | 4.6830606 | -2.03 | FNAGPRICLG |
| Apaniculata113504_c0_seq1_len=757 | >CYP86A96 | 210 | 6.11 | 23191.78 | 233 | 1.62658 | 0.7018426 | -6.02 | FNAGPRICLG |
| Apaniculata50720_c0_seq1_len=1932 | >CYP86B29 | 518 | 6.25 | 60366.96 | 1105 | 3.03 | 1.6 | -4.79 |  |
| Apaniculata14982_c0_seq2_len=1688 | >CYP87A40 | 200 | 8.95 | 23010.75 | 171 | 0.53535 | -0.901441 | -7.62 |  |
| Apaniculata15244_c0_seq1_len=2551 | >CYP87A41 | 135 | 9.54 | 22454.9 | 292 | 0.61 | -0.72 | -7.1 |  |
| Apaniculata111421_c0_seq1_len=1229 | >CYP87E4 | 167 | 5.88 | 18714.6 | 160 | 0.68799 | -0.539535 | -7.26 |  |
| Apaniculata34220_c0_seq1_len=1791 | >CYP90A40 | 483 | 9.37 | 54930.49 | 3476 | 10.2565 | 3.3584687 | -3.36 | FSGGPRRCPG |
| Apaniculata21906_c0_seq3_len=1606 | >CYP90B28 | 430 | 9.42 | 49608.44 | 2125 | 7 | 2.85 | -3.58 |  |
| Apaniculata11484_c0_seq1_len=2064 | >CYP90C21 | 493 | 8.97 | 56159.43 | 6469 | 16.5632 | 4.0499057 | -2.67 | FGGGQRLCPG |
| Apaniculata2804_c0_seq1_len=1798 | >CYP92A121 | 508 | 8.37 | 60468.53 | 1899 | 5.59 | 2.49 | -3.91 | FGSGRRMCPG |
| Apaniculata26402_c0_seq1_len=1925 | >CYP94A59 | 508 | 9.26 | 58774.72 | 12519 | 34.37 | 5.11 | -1.29 |  |
| Apaniculata22533_c1_seq21_len=6227 | >CYP94B56 | 524 | 9.16 | 58381.08 | 2798 | 2.37457 | 1.2476646 | -5.47 |  |
| Apaniculata22849_c0_seq18_len=2103 | >CYP94C78 | 510 | 8.99 | 57101.77 | 20335 | 51.1 | 5.68 | -0.71 |  |
| Apaniculata29907_c0_seq1_len=1931 | >CYP94D59 | 505 | 9.23 | 61841.41 | 6560 | 17.96 | 4.17 | -2.22 | FHAGPRMCLG |
| Apaniculata85573_c0_seq1_len=2129 | >CYP94F8 | 516 | 8.36 | 58724.46 | 757 | 1.88 | 0.92 | -5.48 | FHCGPRMCLG |
| Apaniculata52147_c0_seq1_len=1395 | >CYP96A106 | 427 | 6.01 | 50172.72 | 991 | 3.76 | 1.92 | -4.48 |  |
| Apaniculata62700_c0_seq1_len=1891 | >CYP96A96 | 514 | 8.72 | 59485.89 | 731 | 2.04287 | 1.0305999 | -5.69 | FNAGPRTCLG |
| Apaniculata270380_c0_seq1_len=675 | >CYP96A97 | 219 | 5.82 | 25369.12 | 49 | 0.38363 | -1.382228 | -8.1 |  |
| Apaniculata124712_c0_seq1_len=1681 | >CYP96W1 | 499 | 7.31 | 57201.84 | 388 | 1.22 | 0.29 | -6.1 | FNAGPRTCLG |
| Apaniculata18928_c0_seq1_len=2276 | >CYP97B35 | 584 | 7.58 | 65443.4 | 5784 | 13.4299 | 3.7473729 | -2.97 | FGGGPRKCVG |
| Apaniculata8330_c0_seq1_len=2171 | >CYP97C29 | 546 | 6.54 | 60708.92 | 9231 | 22.4701 | 4.4899331 | -2.23 | FSGGPRKCVG |
| Apaniculata19923_c1_seq1_len=1816 | >CYP98A89 | 511 | 8.39 | 59784.07 | 23374 | 68.02 | 6.09 | -0.3 | FGAGRRICPG |
