# Supplementary material for: De novo Assembly of Leaf Transcriptome in the Medicinal Plant Andrographis paniculata
Source: Front Plant Sci. 2016 Aug 17;7:1203. doi: 10.3389/fpls.2016.01203 (PMC4987368; doi:10.3389/fpls.2016.01203)
Supplement: Supplementary File S9 — Summary of simple sequence repeats identified in annotated transcripts. [file Table9.docx]

| Total number of sequences examined: | 83800 |
| --- | --- |
| Total size of examined sequences (bp): | 88933560 |
| Total number of identified SSRs: | 32341 |
| Number of SSRs with mononucleotide repeats | 9139 |
| Number of SSRs with dinucleotide repeats | 12094 |
| Number of SSRs with trinucleotide repeats | 9599 |
| Number of SSRs with tetranucleotide repeats | 1377 |
| Number of SSRs with pentanucleotide repeats | 132 |
| Total number of SSR containing sequences: | 23168 |
| Number of sequences containing more than 1 SSR: | 6540 |
| Number of SSRs present in compound formation: | 2438 |

**Supplementary File S9. Summary of simple sequence repeats identified in annotated transcripts**
