# Supplementary material for: De novo Assembly of Leaf Transcriptome in the Medicinal Plant Andrographis paniculata
Source: Front Plant Sci. 2016 Aug 17;7:1203. doi: 10.3389/fpls.2016.01203 (PMC4987368; doi:10.3389/fpls.2016.01203)
Supplement: Supplementary File S10 — Advantages of present transcriptome analysis over the earlier work published. [file Table10.docx]

**Supplementary File S10. Advantages of present transcriptome analysis over the earlier work published**

| **S.No.** | **Parameter** | **Present investigation** | **Garg et. al. 2015** |
| --- | --- | --- | --- |
|  | Generated transcripts for leaf tissue | 83,800 | 69,011 |
|  | N50 | 1880 bp | 926 bp |
|  | Leaf transcript average length | 1599 bp | 667 bp |
|  | Annotated leaf transcripts | 49,363 | 40,586 |
|  | KEGG annotated leaf transcripts | 5,606 | 4,044 |
|  | KOG annotated leaf transcripts | 35,401 | --- |
|  | Unique KOG mapped leaf transcripts | 14,720 | --- |
|  | TF families assigned | 97 | 22 |
|  | No. of leaf transcripts with SSRs | 23,168 | 13,805 |
|  | CYP 450s identified | 124 ( in leaf only) | 188 ( in both leaf and root) |
|  | Terpene biosynthesis related transcripts | 146 ( in leaf only) | 48 ( in both leaf and root) |

Detailed analysis of CYP450s clearly demonstrates the advantage of present investigation.

| **Range** | **Number of transcripts in the Present investigation** | **Number of transcripts in the Garg et. al. 2015** |
| --- | --- | --- |
| 200-300 bp | 4 | 29 |
| 301-400 bp | 3 | 26 |
| 401-500 bp | 3 | 13 |
| 501-600 bp | 3 | 8 |
| 601-700 bp | 8 | 17 |
| 701-800 bp | 3 | 13 |
| 801-900 bp | 4 | 21 |
| 901-1000 bp | 6 | 12 |
| >1000 bp | 90 | 49 |
| Total | 124 | 188 |
